# Polycyclic aromatic hydrocarbons and perinatal outcomes: a scoping review

**DOI:** 10.1038/s41390-026-05264-1

**Published:** 2026-07-03

**Authors:** Srirupa Hari Gopal, Melissa A. Suter, Bhagavatula Moorthy, Mohan Pammi

**Affiliations:** 1 Dept. of Pediatrics, Division of Neonatology, SSM Health Cardinal Glennon Children’s Hospital/Saint Louis University, St Louis, MO USA; 2 Dept. of Obstetrics and Gynecology, Baylor College of Medicine, Houston, TX USA; 3 Dept. of Pediatrics, Division of Neonatology, Baylor College of Medicine & Texas Children’s Hospital, Houston, TX USA

## Abstract

**Abstract:**

Polycyclic aromatic hydrocarbons (PAHs) are generated during the incomplete combustion of carbon-based materials and have been identified as mutagenic, teratogenic, carcinogenic, and immunotoxic agents. Maternal exposure to PAHs via inhalation, occupational exposures, dietary intake, or dermal absorption has been linked to an elevated risk of infertility, spontaneous abortion and preterm birth. PAHs can cross the placenta, potentially leading to adverse fetal outcomes including growth restriction and congenital anomalies. Additionally, both prenatal and postnatal exposure to PAHs have been associated with a range of childhood disorders, including asthma, attention deficit hyperactivity disorder, autism spectrum disorders, pediatric cancers, and allergic conditions. The objective of this scoping review is to provide a comprehensive overview of current evidence regarding the effects of PAH exposure on maternal, fetal, neonatal, and childhood health outcomes.

**Impact:**

- Polycyclic aromatic hydrocarbons (PAHs) are hazardous organic pollutants that have been implicated in adverse maternal, fetal, neonatal and childhood outcomes.
- Appreciation of the mechanisms, exposure pathways, and their correlation with health outcomes can facilitate the development of informed policies and effective strategies for exposure management, prevention and/or treatment of diseases associated with PAH exposure.
- This scoping review summarizes current literature on the impact of PAH exposure on perinatal and childhood health outcomes.

## Introduction

Exposure to environmental pollutants has been implicated in human diseases. Polycyclic aromatic hydrocarbons (PAHs) are a class of hazardous organic compounds considered as environmental pollutants. PAHs are specifically emitted from the process of incomplete combustion of carbon sources such as vehicles, coal burning units, and gasoline derivatives. They are also prevalent in tobacco smoke and forest fires. Cooking certain protein-rich foods at high temperatures such as meat releases PAHs.^1–3^ Recent studies have reported the presence of PAHs in the context of hydraulic fracturing to produce oil and gas from geological formations. Fifty-four PAHs have been identified at National Priorities List hazardous waste sites, also known as Superfund sites. Of these, 16 have been designated as priority pollutants by both the U.S. Environmental Protection Agency and the European Commission due to their environmental persistence, ubiquity and documented harm to human health.^4^

PAHs can be categorized as either “light” or “heavy.” The light PAHs, with up to four fused benzene rings, are generally more volatile and exist more commonly in the gaseous phase as atmospheric vapors, whereas the heavy PAHs, containing more than four rings, are less soluble but are more persistent and toxic (Table 1).^4–6^ The heavy PAHs typically exist in both vapor and particulate matter. PAHs have been reported to exhibit multiple adverse effects to human health through the induction of oxidative stress, leading to carcinogenicity, immunotoxicity and teratogenicity.^1,2^ Epidemiological studies have reported concentrations of PAHs in humans using various human tissues and fluids (blood, urine, placenta) in different parts of the world, revealing geographic diversity with regards to exposure levels and tissue concentrations.^4^

**Table 1:** Categories of polycyclic aromatic hydrocarbons (PAH)

Categorization of Polycyclic Aromatic Hydrocarbons	
Light (≤ 4 rings)	Heavy (> 4 rings)
Napthalene	Benzo(a)pyrene
Phenanthrene	Benzo(g,h,i)perylene
Anthracene	Indeno(1,2,3-c,d)pyrene
Benzo(a)anthracene	Benzo(k)fluoranthene
Benzo(b)fluoranthene	Dibenz(a,h)anthracene
Acenapthylene	Acenaphthene
Fluorene	
Fluoranthene	
Pyrene	
Chrysene	

Sixteen high priority PAHs^4–6^.

*PAHs* Polycyclic Aromatic Hydrocarbons.

## Mechanism of PAH toxicity

PAHs are released into the atmosphere, following their formation, and then deposited in soil, sediments, plants and water, through atmospheric transport, dry deposition and wet deposition. PAHs are highly lipophilic (3–8 log K˳w), which allows them to easily accumulate in living organisms, especially in fat tissue. Aqueous solubility decreases for each additional ring added to the PAHs. Humans are exposed to these persistent compounds through inhalation, ingestion and absorption. Inhalation of pollutants mainly comes from products of incomplete combustion of fossil fuels such as car exhaust, smoke from forest fires or first or secondhand tobacco smoke. Ingestion of PAHs occurs through drinking contaminated water and the ingestion of grilled meats. Exposure can even occur through dermal absorption after contact with polluted soils.^3^

Biotransformation is a key factor in determining the toxicity of PAHs and their derivatives.^3,5^ In their original form, most PAHs are relatively inactive and do not directly cause biological damage. However, once inside the body, they undergo metabolic activation, primarily through enzymes such as cytochrome P450 (CYP)^3^, that exist in multiple isoforms (e.g., CYP1A1, 1A2, 1B1) with overlapping substrate specificities.^7–10^ Metabolic activation converts PAHs into reactive intermediates, including diol-epoxides and o-quinones. These metabolites can interact with cellular macromolecules, binding to DNA and proteins and forming adducts that disrupt normal cellular processes. Such interactions may result in genetic mutations, structural damage, and interference with essential protein functions.^3,5^ In addition, reactive PAH metabolites can generate oxidative stress, which further contributes to cellular dysfunction, inflammation, and the initiation of signaling pathways that may ultimately lead to programmed cell death. Whether inhaled, ingested or absorbed, upon exposure, these PAHs are quickly converted to their hydroxylated metabolites. These hydroxylated metabolites serve as biomarkers for PAH exposure and to explore associations and causal relationships between PAH exposure and disease processes.^5^

Pregnancy is unique in that, PAHs have the potential to affect both maternal and fetal health since PAHs can easily cross the placenta, affecting the fetus in the crucial phases of growth and development.^2^ There is extensive scientific evidence on the endocrine disruption that PAHs cause to maternal health, due to their anti-estrogenic and anti-androgenic properties.^1,11–14^ These components have been implicated in several maternal, perinatal and neonatal outcomes such as pregnancy loss, fertility, preterm delivery, fetal growth and neonatal health and wellbeing. In addition, due to the potential to elicit epigenomic modifications, PAHs have also been implicated in long-term outcomes such as child neurobehavioral development, childhood cancers and childhood respiratory illness.^15–18^

**Fig. 1:**
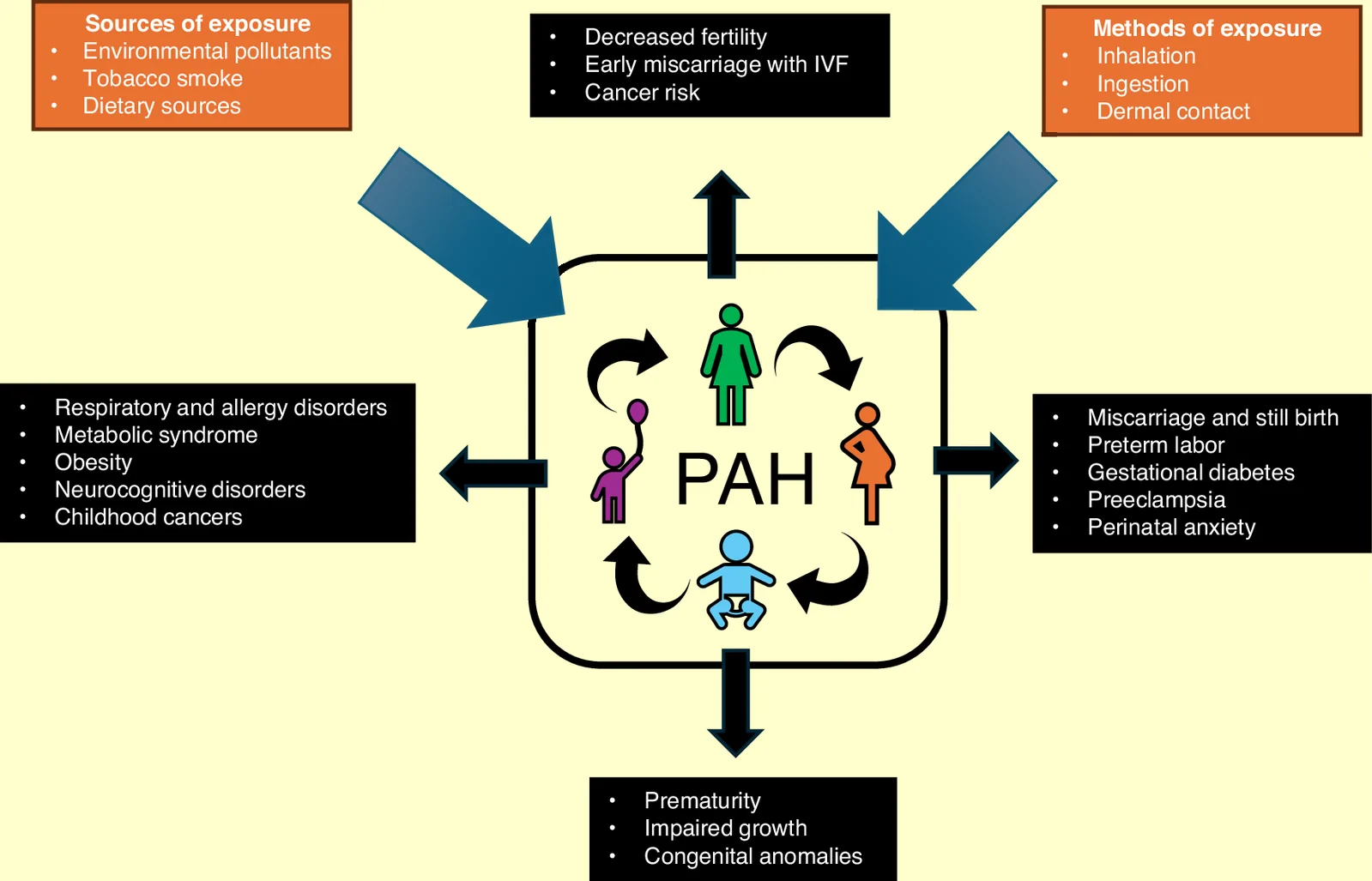
PAH exposure and childhood outcomes. Schematic showing maternal PAH exposure and its impact of maternal, fetal, neonatal and childhood outcomes.

A schematic describing the effects of PAH on maternal, perinatal, neonatal and childhood outcomes is shown in Fig. 1.

## Biomonitoring for PAH exposures

When assessing environmental health literature for outcomes, it is important to understand how personal exposures are inferred. Scientists and epidemiologists use multiple modalities to understand the burden and timing of exposures in human health. With regards to pregnancy, timing of exposure is critical in our understanding as to “how much” and “when” exposures to PAHs affect pregnancy and neonatal outcomes. In general, studies of PAH exposures utilize three different ways to assess exposures: (1) quantitative measurements in biological samples; (2) personal device monitoring and (3) geostatistical modeling incorporating information on residence and the levels of PAHs in the atmosphere over time.

### Quantitative measurements in biological samples

As noted, after exposure to PAHs, the CYP P450 enzymes metabolize the parent PAH compound to an excretable metabolite. Therefore, measurements of exposures need to take into account not only the PAHs of interest, but also the known metabolites. For example, 3-hydroxybenzo[a]pyrene (3-OH-BaP) is a well characterized BaP metabolite that can be readily detected in urine, and is used as a biomarker of BaP exposure. Urinary PAH metabolites yield insight into recent exposures, as PAHs are rapidly metabolized and eliminated through the urinary system.^19^ If blood or tissue samples are available, PAHs can be extracted from the blood or tissue using organic solvents. Gas chromatography followed by mass spectrometry (GC-MS) can be then used to quantify levels of the parent PAH compound. Furthermore, PAHs are able to form bulky adducts through covalent bonds on the DNA backbone. These adducts can be measured using GC-MS or thin layer chromatography techniques and reflect exposure over longer periods of time compared with urinary biomarkers.^20,21^

### Personal device monitoring

Prospective studies of environmental exposures may use personal device monitoring which allows for personalized assessment of chemical exposures over a period of time. Studies utilize small backpacks containing air monitoring devices that a subject will wear for a defined period of time each day.^22^ Other studies employ the use of silicone wrist bracelets which absorb organic compounds.^23^ These are worn daily for a specified amount of time after which the organic pollutants can be extracted from the wristband and analyzed via GC-MS.

### Geostatistical modeling

Some epidemiologic studies estimate exposure to polycyclic aromatic hydrocarbons (PAHs) as well as many other compounds including particulate matter by applying geostatistical models that combine publicly available air monitoring data, emissions data, and information of area of residence. Such modeling allows researchers to assign exposure estimates even in areas without direct measurements.^24^

## Gaps in current literature

Despite a growing body of evidence on the associations between PAH exposure and maternal health, a comprehensive evaluation that traces PAH exposure effects across the continuum from pregnancy to childhood has not been performed. The current literature remains vast and fragmented, challenged further by significant heterogeneity in exposures, methods used, confounding variables and biomonitoring practices. While reviews on specific domains such as fertility and reproductive health, birth outcomes, endocrine disruption, neurodevelopment, or childhood respiratory disease have served as a valuable guidance, examining the continuum of effects from maternal exposure during pregnancy through childhood is vital to understand the whole spectrum of effects of PAH on maternal, perinatal and childhood outcomes. Uncertainties continue to remain regarding critical windows of vulnerability during fetal development and early childhood, dose-response relationships, and the relative contribution of individual PAH compounds versus complex environmental mixtures. This scoping review aims to fill that gap by providing an in-depth assessment of toxicological evidence, mapping the impact of PAH exposure on maternal, perinatal, neonatal, and subsequent childhood health.

**Objectives**

1. To map and summarize the evidence on PAH exposure during pregnancy and postnatally and its impact on fetal and neonatal and childhood outcomes.
2. To identify knowledge gaps and inform future research.

## Methods

We performed a scoping review according to established guidelines.^25^ We utilized the following steps (a) developed research questions, (b) identified relevant studies, (c) selected studies, (d) charted the data, and (d) collated, summarized and reported the findings.^25^

### Search strategy

We searched the following electronic databases on September 18, 2025 (Appendix 1):

1. MEDLINE, Cumulative Index to Nursing and Allied Health Literature (CINAHL) through EBSCOhost, Embase, and the Cochrane Central Register of Controlled Trials (CENTRAL) in the Cochrane Library.
2. PubMed’s related citations feature was used to identify relevant articles. Additional research was made from articles cited by the included studies and by contacting experts in the field.

### Inclusion criteria

We included prospective or retrospective, cohort or cross-sectional studies that reported on PAHs and maternal, perinatal, neonatal and childhood outcomes. Only articles published in English language were included.

### Selection and data collection process

**Fig. 2:**
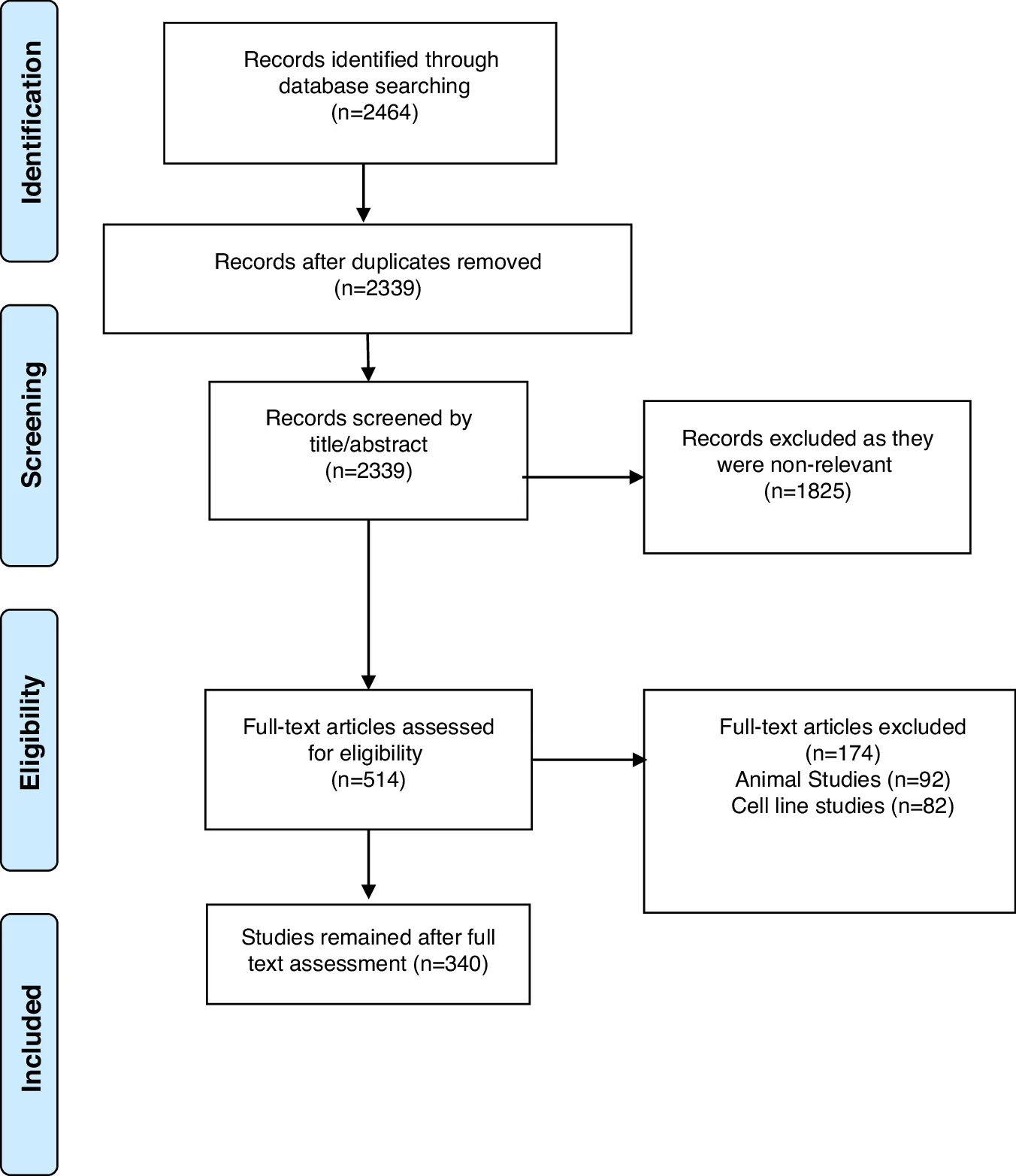
PRISMA flow diagram of included studies. We identified 2339 records through database searches, and the process of inclusion is detailed in the figure. We included 340 studies for our qualitative summary of the literature pertaining to our objectives.

Articles were screened for eligibility by two authors (MP and SHG). We identified 2339 records through database searches. The inclusion process is detailed in the PRISMA flow diagram (Fig. 2). We included 340 human studies for our mapping of studies on PAHs exposure and outcomes. The included studies were thereafter divided into pertinent categories related to: maternal health (fertility, fetal viability, pregnancy associated complications), prenatal and postnatal exposure to PAH and its impact on neonatal and childhood outcomes. A summary of the existing evidence on various included outcomes is shown in Table 2.

**Table 2:** Summary of evidence on various maternal, neonatal and childhood outcomes following prenatal and postnatal PAH exposures.

Outcomes	Summary of Evidence
Pre-conceptional health and fertility	PAH exposure→ ↓ female and male fertility^1,26^
In-vitro fertilization success	↑ PAH levels→ ↓ IVF success and ↑ miscarriage risk^27,30^
Pregnancy related disorders	Air pollution, toxic metal exposure and occupational chemical exposure → ↑ risk for gestational diabetes and pre-eclampsia^54,55^ PAH→ ↑ risk of perinatal anxiety^61^
Preterm birth	*Conflicting results in systematic reviews* Prenatal PAH exposure→ ↑ risk for preterm birth^62^
Neonatal anthropometrics	prenatal PAH exposure→ ↓ birth weight, ↓birth length and ↓ head circumference Effects vary with sex and race^2,80,109^
Congenital anomalies	Prenatal PAH exposure→ ↑ risk for neural tube defects,^81–83^ oral cleft,^85,86^ and congenital heart defects^87^
Childhood respiratory and allergy disorders	Prenatal and postnatal PAH exposure→ ↑ allergy, asthma and respiratory symptoms Effects vary with sex^19,145,157,187^
Childhood neurocognitive disorders	Prenatal and postnatal PAH exposure→ ↑ ADHD, Autism, anxiety and depression Effects vary with sex^162,166,173,190^
Childhood metabolic and endocrine disorders	Prenatal and early-life PAH exposure → ↑ risk for altered growth trajectories, childhood obesity^180,182,187^ PAH exposure→ ↑ blood pressure, ↑ risk for metabolic disorders, and ↑ risk for delayed puberty in girls.^189^

Polycyclic Aromatic Hydrocarbons-PAH, In-vitro fertilization- IVF, Attention deficit hyperactivity disorder (ADHD).

#### PAH exposure and maternal health outcomes

The female reproductive system is noted to be particularly vulnerable to environmental chemical exposures, in part since oocytes are the longest-lived non-regenerating cells in the body and thus subject to a lifetime of chemical exposures. This extended period of susceptibility means that harmful substances, such as PAHs, can accumulate and potentially disrupt reproductive health, affecting fertility and pregnancy outcomes.^1^ Furthermore, as PAHs can cross the placental barrier, maternal exposure results not only in direct effects on the mother but also initiates the exposure-disease process in the offspring.

### PAH exposure and fertility

PAHs have been reported to be endocrine disrupting chemicals (EDCs), having anti-estrogenic and anti-androgenic properties; EDCs have the potential to affect reproductive endocrine pathways, fertility, pregnancy outcomes, and general maternal health, as well as increase the risk of cancer. From the fertility standpoint, PAHs have been shown to cause oxidative damage, inflammatory responses, immune damage and DNA mutations in the testes and ovaries, which may subsequently contribute to infertility.^1,26–28^

#### PAH exposure and female fertility

Evidence of the harmful effects of PAHs on female fertility has been shown in studies demonstrating the impact of PAHs on ovarian follicular health and on in vitro fertilization (IVF) success rates. Combustible tobacco smoke contains high levels of PAHs, and smokers require almost twice as many IVF cycles compared with non-smokers to achieve a successful pregnancy.^27^ Benzo(a)pyrene (BaP) impairs cell proliferation, inhibits estrogen production, and contributes to DNA damage. Li et al. showed that concentrations of urinary PAH-metabolites can potentially be used as biomarkers to predict early miscarriage during IVF-embryo transfer.^29^ Multiple studies of follicular fluid correlate PAHs and other environmental contaminants. Increased BaP levels were also demonstrated in the follicular fluid of smokers undergoing infertility treatment, and higher BaP levels were found in the smokers who did not conceive compared to those who had achieved pregnancy.^30^ Further, PAHs exposures have also been investigated in the context of IVF success.^29,31^ In a large study assessing multiple environmental chemicals in follicular fluid, PAHs were detected in 100% of all samples tested (*N* = 88).^32^ Another study showed a difference in levels of bispohenol a in the follicular fluid based on residence. Specifically, individuals living in a high environmental impact area had significantly higher bisphenol A levels in follicular fluid compared with women from a low environmental impact area.^33^

### PAH exposure and male fertility

PAH exposure in males also affects fertility and pregnancy outcomes. Increasing evidence indicates that semen quality represents a sensitive and early biomarker of environmental exposures. Spermatozoa are vulnerable to environmental pollutants due to their limited DNA repair capacity and high susceptibility to oxidative stress. ^34^ Recent studies demonstrate that pollutant exposure is associated with increased reactive oxygen species (ROS) generation, leading to lipid peroxidation, DNA fragmentation, and impaired sperm function. ^34^ Two studies specifically assessing PAHs found that men with higher PAH exposures exhibited increased oxidative stress, reduced antioxidant defenses, and significantly elevated sperm DNA fragmentation, indicating a strong link between PAH exposure and impaired sperm integrity.^35,36^ Newer methods such as volatilomics-based semen profiling have been investigated as a non-invasive and innovative method to assess sperm quality and may help elucidate environmental exposures, oxidative stress and PAH mediated alterations affecting male fertility.^37^

### PAH exposure and risk of still birth and miscarriages

Exposure to PAHs can disrupt placental, hormonal, and follicular functions, increasing the risk of pregnancy loss. PAHs may impair trophoblast proliferation and cause embryonic damage, contributing to miscarriage and stillbirth. Numerous studies have linked air pollution, a major source of PAH exposure, to early pregnancy loss, providing important evidence of its reproductive impact.^38–47^ Furthermore, tobacco smoke exposure, which contains more than 15 PAHs is associated with increased risk of both stillbirth and miscarriage.^48–50^

### PAH exposure and general maternal health

PAH exposure and maternal-fetal outcomes have been studied in the context of two common pregnancy complications, namely gestational diabetes mellitus (GDM) and preeclampsia (PE). Preeclampsia is believed to stem from abnormal placental pathology, indicating a potential role of toxin exposure in the development of PE.^51,52^ Yet, tobacco smoking, which is a known source of PAHs, has not been established as a risk factor for GDM, and paradoxically, has been reported to be protective for the development of PE.^53^ However, air pollution has known associations with both GDM and hypertensive disorders of pregnancy (HDP) such as PE.^54–57^ This was further investigated involving occupational exposure to chemicals including lead, motor exhaust and organic pollutants, with increased association with GDM and HDP.^53,58^

Exposure to PAHs has been reported to be associated with increased oxidative stress during pregnancy.^59^ Exposure to local traffic-generated air pollution during pregnancy has also bene reported to increase the risk of preeclampsia.^60^ In addition to GDM and HDP, other outcomes associated with PAHs include blood coagulation during pregnancy, with urinary PAH levels associated with increased fibrinogen and shortened coagulation time, thus increasing the risk of pregnancy associated with thrombotic complications.^57^ A strong positive link has also been reported between PAH exposure and perinatal anxiety, higher urinary concentrations of PAH components were reported to be associated with perinatal anxiety (OR = 2.055, 95%CI: 1.355-3.117).^61^

### PAH exposure and preterm birth

Preterm labor resulting in spontaneous preterm birth (PTB) is a global problem with high infant morbidity and mortality. ^62^While etiology remains elusive, many modifiable and non-modifiable risk factors are associated with increased risk. Epidemiological studies have reported that maternal exposure to air pollution, tobacco smoke, ambient environmental chemicals, PAHs, and toxic metals increase the risk of preterm delivery.^62^ Placental transfer of these pollutants can result in preterm delivery due to various mechanisms including inflammatory damage, oxidative damage, DNA damage and epigenetic changes.^62^ Recognition of this hazardous exposure and its effects is important to prevent adverse outcomes in the maternal-fetal dyad.

Measurement of PAH metabolites in maternal urine is important to determine maternal exposure levels and measurement in placental tissue or cord blood can provide insight into maternal and fetal exposures. Some prospective cohort studies have leveraged personal device monitoring during pregnancy to determine levels of PAHs in ambient air, while others have used geographical data to determine levels of PAH exposures based on residence and levels of air pollution.^20,63^ Using gas chromatography, mass spectrometry, absolute levels of individual PAHs in placenta tissues can be measured. All of the 16 EPA priority PAHs have been found in placenta tissue and the presence of individual PAHs as well as levels detected can vary greatly based on location.^51^ Placental benzo(b)fluoranthene (BbF) levels were found to be inversely correlated with gestational age at delivery (r(s) = −0.171, *p* = 0.002). Additionally, placental dibenz(a,h)anthracene (DBA) concentrations were higher in placentae from spontaneous preterm births compared to those from medically indicated preterm births (mean 743.7 vs. 599.9 ng/mL, *p* = 0.049), indicating a possible association with spontaneous preterm birth.^64^ Higher levels of BaP and malondialdehyde (MDA), were observed in placentae from women who delivered preterm compared to those who delivered full-term, demonstrating a possible association of PAH with PTB.^65^ A recent systematic review on environmental pollutants and preterm birth summarized the existing evidence on this topic and reported conflicting evidence.^62^ Included studies showed a significant association between air pollutants, particulate matter, PAHs, persistent organic compounds, tobacco smoke, toxic chemicals and global warming with adverse pregnancy outcomes such as preterm birth, fetal growth restriction, risk of PPROM and adverse gestational outcomes. Due to conflicting results and heterogeneity, a meta-analysis was not feasible.^62^

In a cohort study involving healthy, nonsmoking women wearing personal monitoring devices to monitor ambient air exposures, preterm delivery risk was 5-fold greater among African Americans with increasing level of PAH exposure.^66^ These exposures were also associated with lower birthweight and decreased head circumference.^67^

A study, which utilized spatio-temporal analyses, investigated PAHs concentrations that mothers were exposed to, based on distance from a primary air pollution monitoring site in Fresno, CA.^68^ Pregnancy outcomes of women who resided within 20 Km of the monitoring site were studied, and address of primary residence was used to calculate exposures. In this study they found an association between PAH exposure during the 6 weeks before delivery and early preterm birth.^68^ Other studies using emissions data from household pollution, local traffic related air pollution, and aircraft emissions, all of which have high levels of PAHs, have also been reported to have associations with PTB.^60,69–71^

Barrett et al. leveraged the CANDLE cohort, which includes 812 subjects, to determine how PAHs influence pregnancy outcomes.^72^ They showed a modest association between hydroxylated PAHs (OH-PAHs), particularly 2-OH-Phenanthrene(2-OH-PHEN), and levels of placental corticotropin releasing hormone (pCRH), which plays a role in parturition.^72^ Urinary concentrations of PAH-metabolite levels taken in pregnancy can help understand timing of exposure in pregnancy and pregnancy outcome. Urinary concentrations of PAHs measured from pregnant women in the PROTECT birth cohort showed that most PAHs were linked to earlier delivery and increased odds of PTB.^73^ Notably, 2-hydroxynapthalene (2-NAP) was significantly associated with increased overall PTB risk (OR: 1.55; 95% CI: 1.05–2.29). For female fetuses, 2-NAP raised overall PTB odds (female OR: 1.52; 95% CI: 1.02–2.27), while no significant association was found for males.^73^ This was also confirmed by Freiji et al. who had reported that significant inverse associations between 1-hydroxypyrene [1-OH-PYR] and PTB (OR: 2.65 [95% CI: 1.39, 5.05]); and 2-hydroxynaphthalene [2-OH-NAP]with GA: -2.46 days [95% CI: −4.15, −0.77]), thus establishing a sexual dimorphic response to PAH exposures and an increased risk of PTB.^74^

Increased activity of placental Aryl Hydrocarbon Hydroxylase (AHH), induced by many PAHs, may be an important determinant of human fetotoxicity. A significant increase in prevalence of placental AHH induction with threatened preterm labor was shown (OR 2.8; 95% confidence interval [1.3–6.2]; chi 2 = 6.7 *p* < 0.01).^75^ While multiple studies, as noted above, have found evidence for a role of PAHs in preterm delivery,^45,55–57^ reports of associations between PAH and PTB remain inconsistent, as many studies have not found an association between exposure and PTB.^76–79^ Notably, Zhao et al. additionally noted that the intake levels of dietary vitamins, carotene, and folate may influence this association across various stages of pregnancy, which may contribute to the variability across these studies.^71^

#### PAH exposure and neonatal health outcomes

The placental transference of PAHs from maternal exposure can impose a significant health risk to the developing fetus, with long term implications. Various outcomes have been explored, and associations have been reported between maternal PAH exposure and neonatal outcomes. The largest and the most heterogenous body of evidence is on the effect of PAH exposure and fetal growth, small for gestational age, low birth weight and other postnatal anthropometric measurements.^2,80^ In addition, the association between maternal PAH and development of neonatal congenital defects such as neural tube defects,^81–84^ oral cleft,^85,86^ and congenital heart defects.^87^ have been explored, with reports of increased incidence of congenital defects with prenatal PAH exposure. Of note, PAHs with their associated risk for preterm birth have been investigated as a potential risk factor for bronchopulmonary dysplasia (BPD), with recent evidence of increased susceptibility to hyperoxic lung injury in animal models with prenatal PAH exposure.^88^ Neonates may be exposed to PAHs both prenatally, via maternal exposure and placental transfer, and postnatally via environmental pollutants^89^ such as tobacco smoke, and, from the mother via breast milk.^90,91^

### PAH exposure and epigenetic changes

One way that PAH exposures experienced in utero can have a long lasting effect is through epigenetic changes to the exposed offspring. These are stable changes to the expression profile of genes without changes to the underlying DNA sequence and include DNA methylation and histone modifications. While the implications of such changes are unclear, a few studies have shown associations between maternal exposures, epigenetic changes and offspring outcomes. A study of 400 pregnant women and their newborns assessed levels of 5-hydroxymethylcytosine (5-hmC), an epigenetic modification, in cord blood as well as maternal urinary PAH metabolites.^92^ Children’s neurodevelopment was assessed at birth and at age 2. The findings implicated an association between higher levels of maternal PAHs with lower scores on the Gessell developmental score and higher cord 5-hmC levels. Another study of 75 pregnant assessed urinary biomarkers, DNA methylation patterns in cord blood and mononuclear cells and placental tissue.^93^ They found that higher levels of specific PAH metabolites (e.g., 1‑hydroxynaphthalene, 1‑hydroxypyrene) were associated with differential CpG methylation in the placenta, with consistent signals observed for 1‑hydroxynaphthalene.

### PAH exposure and the potential for transgenerational inheritance

Alterations to the epigenome can have a long lasting effect. For example, in a cohort of 302 men, higher urinary PAH metabolites were associated with altered DNA methylation at imprinted genes (H19, Peg3, and Meg3) in sperm.^94^ Alterations of the paternal epigenome in sperm cells is a heritable mark associated with PAH exposure. Furthermore, paternal PAH exposure was also inversely associated with newborn birth weight and increased methylation of the imprinting gene H19 was specifically linked to lower birth weight, indicating a potential epigenetic mechanism through which paternal PAH exposure influences offspring outcomes. A study in a murine model also implicates that PAH-induced epigenetic changes may persist across generations. In a mouse model, prenatal exposure to PAHs led to increased body weight, fat mass, and adiposity-related gene expression in offspring, accompanied by reduced DNA methylation of the PPARγ gene, a key regulator of adipocyte differentiation. ^95^ These metabolic and epigenetic alterations persisted into the grand-offspring generation, indicating multigenerational effects of in utero exposure. Together, these studies provide evidence that PAH exposure may induce durable epigenetic alterations.

### PAH exposure and neonatal growth parameters

There is extensive literature on the impact of maternal PAH exposure prenatally and its effect on fetal growth, and subsequently birth weight.^2,96–98^ Latifi et al. performed a systematic review and metanalysis on the association between prenatal PAH exposure and birth anthropometric outcomes, with report that maternal exposure to PAHs was associated with adverse birth anthropometric outcomes, particularly lower birth weight and birth length.^2^ PAHs have been studied in different samples including placenta,^99–105^ umbilical cord^24,106–111^ and meconium.^112–114^ Agarwal et al. reported an inverse relationship between PAHs and birth weight of neonates as noted in the placental samples.^99^ This effect of prenatal exposure and fetal growth impact has been reported in a wide range of exposures including dietary sources, ^115–118^ wildfire smoke,^119^ smoke from kerosene cooking,^120^ combustion of coal,^121^ industrial smoke,^122–125^ occupational exposures,^126^ and air pollution.^11,43,44,118,127–132^ It has also been reported that the impact of PAHs on fetal growth is most prominent in the first trimester.^133^ Prenatal exposure to PAHs may be associated with an increased risk of low birth weight in infants by affecting DNA methylation states of genomic DNA and growth-related genes (IGF1 and IGF2) in umbilical cord blood. This suggests that reducing environmental PAHs exposure could help improve infant health outcomes.^106^

The impact of birth weight from prenatal PAH exposure has also been reported to exhibit variability based on sex, gender, race and ethnicity. For instance, in a study by Choi et al., prenatal exposure to PAHs was significantly associated with reduced birth weight among Krakow Caucasians and NYC African Americans, after adjusting for known and potential confounders^66,107^. However, this association was not noted in NYC Dominicans.^66,107^ Maternal obesity significantly exacerbated the risk of prenatal PAH exposure in African American newborns. Jedrychowski et al. and Ghosh et al also reported gender differences in fetal growth of newborn exposed to fine particulate matter.^134,135^

Like fetal and postnatal weight, prenatal exposure to PAHs can also have significant impact on the birth length.^132,133^ Ballester et al. reported that prenatal exposure to ambient nitrogen dioxide from air pollution during the first trimester was associated with change in birth length.^132^ An increase in prenatal exposure to fine particles during pregnancy was associated with an average birth weight decrease of 97.2 g, and a reduction in birth length of 0.7 cm.^134^ Sram et al. also reported a possible association between higher concentration of OH-PAH metabolites in newborns’ urine and decreased length.^105^

Despite the growing body of evidence, there are multiple studies with contradicting results, reporting limited or no association between prenatal PAH exposure and birth anthropometric outcomes, with potential sources of heterogeneity, ethnicity, regions, and exposure levels.^41,79,80,96,136,137^ The variability in findings reflects the complexities involved in determining the impact of PAH exposure on SGA outcomes. Differences in population characteristics, methods of measuring exposure, and statistical approaches can all contribute to inconsistencies, making meta-analysis and interpretation within this field more challenging.

### Dietary PAHs and neonatal outcomes

Dietary intake is a major route of human exposure to polycyclic aromatic hydrocarbons (PAHs), largely due to cooking processes such as grilling, smoking, frying, and roasting, as well as from environmental sources entering the food chain.^138^ Studies have specifically interogated PAHs from food sources and neonatal outcomes. Dietary PAH intake was associated with decreases in birth weight by 5.65 g (95% confidence interval [CI]: −16.36, 5.06) and birth length by 0.04 cm (95% CI: −0.08, 0.01), as well as an increase in head circumference (effect size = 0.001; 95% CI: −0.003, 0.005).^118^ Also, frequent dietary consumption of PAH-laden food items posed an independent risk on the reduced birth weight among African Americans.^139^ Additionally, it has been reported that intake of vitamin C during pregnancy may, in fact, reduce the effect of dietary benzo(a)pyrene on birth weight.^115^

### PAH exposure and congenital defects

PAHs have been studied for their association with several birth defects. For instance, tobacco smoke exposure has been implicated in several birth defects, supporting the evidence that PAH exposure can be associated with health implications in neonates.^81–87^ Langlois et al. reported that maternal occupational exposure to PAHs during pregnancy was associated with cleft lip, with or without cleft palate.^85^ This was also supported by Ravi et al., who had reported that smoke exposure resulting from cooking, heating, or smoking (whether direct or passive), as well as direct inhalation of gas, was most strongly associated with cleft palate: 60.7% in unilateral cleft palate, 90.9% in bilateral cases, 65.0% in soft tissue, and 76.2% in hard tissue cleft palate.^86^ Similarly, occupational exposure to PAHs has also been implicated in the development of fetal neural tube defects (NTDs).^81,83^ An elevated placental level of PAHs has been shown to be associated with increased risk of fetal NTDs.^82^ This was also reported with the evidence of PAH-DNA adducts in the cord blood of neonates with NTD.^84^ Further, PAH exposure studied in maternal hair has been reported to be associated with an increased risk of congenital heart defects.^87^ Research on the teratogenic effects of PAHs includes data from a large population-based National Birth Defects Prevention Study, which found an association between maternal occupational exposure to PAHs (in mothers aged 20 or older) and gastroschisis.^140^ Various other birth defects such as craniosynostosis, birth defects of face and central nervous system have also been reported.^141,142^

#### PAH exposures and child health outcomes

Children are especially susceptible to the impacts of environmental hazards, with potential exposures occurring from fetal development onward. The impact of prenatal and postnatal PAH exposures including air pollution, tobacco smoke, and maternal PAH exposure during pregnancy have all been investigated with reports of association with various outcomes such as respiratory and allergy disorders, neurocognitive disorders, cardiac disorders, childhood cancers and metabolic problems including childhood obesity (Table 2).^143–150^

### Respiratory and allergy disorders

There is substantial evidence that environmental pollutants cause respiratory illnesses in children.^151^ PAHs are known to cause inflammation and immunological changes, which can predispose children to reactive airway disease, asthma and allergic symptoms.^152^ PAHs can also cause epigenomic alterations via DNA methylation, thus augmenting the effects of environmental exposures.^145,153^ This cause-effect relationship has been demonstrated in several studies in the context of both prenatal and postnatal PAH exposures. Jedrychowski et al. found that prenatal exposure to immunotoxic PAHs affects respiratory symptoms such as barking cough, wheezing, sore throat, ear infection, and cough, regardless of respiratory infections. Postnatal passive smoking partly modified the effect on runny nose or cough, and exposure also lengthened symptom duration.^154^ The investigators further reported a significant deficit in the lung function of pre-school children of non-smoking mothers, with prenatal exposure to fine particulate matter.^155^ Miller et al. provided additional evidence indicating that early exposure to airborne PAHs, and environmental tobacco smoke (ETS) is associated with an increased risk of respiratory symptoms and likely asthma in children between 12 and 24 months of age.^156^ This prenatal effect of PAH exposure was also reported to have a dimorphic effect based on the sex of the child, with report of adverse respiratory outcomes with prenatal PAH exposure.^19^ Beyond evidence regarding prenatal PAH exposure, studies have also linked postnatal and early childhood exposure to PAHs with an increased risk of early onset wheezing symptoms.^148,157^ Notably, prenatal PAH exposure may not only trigger but also exacerbate the development of childhood wheezing associated with subsequent postnatal exposure.^153^

### PAH exposure and childhood neurocognitive disorders

In addition to being immunotoxic, mutagenic and carcinogenic, PAHs have been reported to be neurotoxic as well. This neurotoxic effect can begin via prenatal exposure, with current evidence reporting prenatal exposure to PAH to be associated with several neurodevelopmental and neurocognitive disorders such as attention deficit hyperactivity disorder (ADHD), autism, anxiety, adolescent behavioral problems, depression and school performance.^21,154,158–169^ Prenatal exposure to particulate matter was also found to be influenced by the sex of the offspring. Studies indicate that higher exposure during specific prenatal periods is associated with reduced memory and attention function, with the strength of these associations varying according to sex.^164,165,170^

Similar effects on neurodevelopmental outcomes have also been reported with postnatal PAH exposure, on various outcomes such as ADHD,^171,172^ autism,^173,174^ dyslexia,^175^ neurocognitive outcomes,^176,177^ neurodevelopmental outcomes,^173,178,179^

### PAH exposure and metabolic and endocrine effects

PAHs are noted to be endocrine disrupting chemicals and as a result, have adverse effects during pregnancy. The mechanism of endocrine disruption is due to negative interactions with various transcription factors, receptors and tissues that result in alterations of metabolic function.^180^ PAHs also exert estrogenic and anti-estrogenic effects, can alter methylation and increase the activity of the primary regulator of adipogenesis, peroxisome proliferator-activated receptor-gamma.^180^ As such, this characteristic of PAHs ought to be considered for several metabolic and endocrine outcomes in children including infant weight trajectories, risk of childhood obesity, metabolic syndrome and menstrual disorders. The effect of prenatal PAH exposure on birth weight has been previously described. This effect has been reported to affect infant growth trajectories.^144,181^ Increased exposure to prenatal PAH has been implicated in obesity and adiposity in children up to the age of 9 years.^182–185^ This was also shown in cohorts with postnatal PAH exposure where higher exposure to early life air pollutants resulted in a higher attained BMI at age 10 years that were independent of later childhood exposures.^186^ Jung et al. found that obese young children experienced higher rates of asthma that were associated with increased exposure to PAHs, specifically methyl phenanthrenes, compared to non-obese children.^187^ Additionally, prenatal exposure to PAHs was reported to be associated with increased systolic, diastolic and mean blood pressures.^188^ The endocrine disrupting ability of PAHs was also explored in the context of pubertal timing, where it was reported that prenatal exposure to PAHs might delay pubertal milestones in girls.^189^

#### Summary and future directions

We report a scoping review mapping the landscape of the adverse impact of PAHs exposure across maternal, fetal, neonatal and childhood outcomes. The interpretation of the precise impact of PAHs on maternal, perinatal, neonatal, and childhood health outcomes is complicated by study heterogeneity, differences in exposure patterns, geographical locations, and the combined effects of multiple PAHs. Furthermore, several confounding variables may influence causal relationships, complicating the extrapolation of results for wider applicability. Despite these challenges, existing literature presents considerable evidence of the adverse effects of PAH exposure on both maternal and fetal health.

Important gaps remain in the current literature base. One of the most important aspects is currently limited is comparatively limited data from low- and middle- income countries, despite higher environmental and occupational exposure burdens. Additionally, data from vulnerable groups belonging to varying socio-economic status, ethnic and cultural groups and underrepresented minority groups is less prevalent. Furthermore, inconsistencies in exposure assessment methods, reporting structure, biomonitoring practices and timing of measurement limit comparability across studies. These findings underscore the need for targeted interventions, including appropriate air pollution regulations, reduced passive and active tobacco smoke exposure, improved regulation of occupational chemical exposures, and enhanced screening and detection of PAHs during pregnancy. Addressing geographical, methodologial and population-level gaps will help improve understanding and generalizability of findings, for future risk assessment, risk-triaging and prioritizing policy development.

### Strengths and limitations

Previous studies have provided a systematic review on the adverse effects of PAHs on maternal reproductive health,^1^ placental function, fetal wellbeing,^4^ neonatal health^41,77^ and childhood neurodevelopmental outcomes individually.^190^ In contrast, the present scoping review aims to examine the scope and breadth of current evidence regarding this topic. Although it does not encompass all existing research, considerable effort has been invested in providing a comprehensive overview of the connections between prenatal exposure and childhood outcomes. Established guidelines for conducting scoping reviews were followed, and included studies were systematically assessed to ensure a thorough summary of the subject matter. The review was limited in not assessing every subtopic for amount, type and time points for exposures, which is beyond the scope of the study. It is important to interpret studies reporting null associations, where substantial heterogeneity in study designs must be considered as a major factor. Many studies differ in their ability to control key confounders, including various co-exposures such as tobacco smoke, ambient air pollution and dietary exposures. This is further challenged by past exposure among the maternal group, which has not been addressed in many studies- which typically have focused on current exposures during pregnancy in a woman. Timing of expsosure, therefore is an impactful factor to taken into consideration while interpreting the available evidence. Furthermore, studies have variability in sample size, population characteristics and underlying exposure distributions, which may partially explain the presence of null findings. Taking into consideration all of these factors, null associations and assumptions have to be interpreted cautiously, taking into account methodical differences, as opposed to definitive evidence of absence of effect. Given the vast evidence base on this topic, a granular evaluation of exposure types, dose-response relationships, and timing of exposure in the entire continuum of maternal, perinatal, fetal and childhood PAH exposures and outcomes, was beyond the scope of this study. This highlights the need for standardized exposure monitoring practices, and outcome assessment for future research.

### Implications for future research

Future research should focus on addressing existing knowledge gaps, including conducting detailed exposure metrics, real time monitoring using innovative, wearable devices, and developing innovative screening techniques for PAHs, such as umbilical cord blood analysis, amniotic fluid testing, maternal buccal swabs, or noninvasive monitoring approaches like transcutaneous testing. Advances in biomarker-based assessment are also needed to improve exposure detection across relevant biological matrices. Additionally, larger-scale studies are warranted to evaluate potential associations between PAH exposure and outcomes across maternal-fetal, neonatal, and childhood stages. Research on biomarker assessment and testing can contribute to drawing informed conclusions and support the development of policies related to air quality, environmental protection, and maternal and child health outcomes.

Improved exposure quantification and biomonitoring could overall help in early screening, and identification of high-risk populations. This is of particular importance to pregnant individuals, who might benefit from close monitoring following exposure, additional imaging to screen for congenital defects, referral to high level of care with advanced obstetric and neonatal care, early on in pregnancy. These advances have important implications for public health policy development, surveillance and implementation of prevention strategies. Understanding the PAH exposure-outcome association may support the development of risk-reduction strategies, including counseling and monitoring for modifiable PAH risk factors such as tobacco smoke, indoor air pollution and PAH exposure from dietary sources. Finally, integrating robust methods to evaluate exposure to clinical and epidemiological research will strengthen ways to facilitate earlier interventions, risk based stratification and ultimately help improve outcomes and protect maternal, neonatal, and childhood outcomes during critical stages of development.

## Supplementary information

Appendix_1.

## References

[1] Bolden, A. L., Rochester, J. R., Schultz, K. & Kwiatkowski, C. F. Polycyclic aromatic hydrocarbons and female reproductive health: a scoping review. *Reprod. Toxicol.* **73**, 61–74 (2017). 10.1016/j.reprotox.2017.07.012 28739294

[2] Latifi, A. M. et al. Association between maternal exposure to polycyclic aromatic hydrocarbons and birth anthropometric outcomes: a systematic review and meta-analysis. *Ecotoxicol. Environ. Saf*. **287**. 10.1016/j.ecoenv.2024.117290 (2024). 39536563

[3] Peng, B. et al. A systematic review of polycyclic aromatic hydrocarbon derivatives: occurrences, levels, biotransformation, exposure biomarkers, and toxicity. *Environ. Sci. Technol.* **57**, 15314–15335 (2023). 10.1021/acs.est.3c03170 37703436

[4] Drwal, E., Rak, A. & Gregoraszczuk, E. L. Review: polycyclic aromatic hydrocarbons (PAHs)-action on placental function and health risks in future life of newborns. *Toxicology* **411**, 133–142 (2019). 10.1016/j.tox.2018.10.003 30321648

[5] Shin, H. M., McKone, T. E. & Bennett, D. H. Evaluating environmental modeling and sampling data with biomarker data to identify sources and routes of exposure. *Atmos. Environ.* **69**, 148–155 (2013).

[6] Fourth national report on human exposure to environmental chemicals: updated tables, January 2019, Volume one. Published online January 1, 2019. 10.15620/CDC75822 (2019).

[7] Elfaki, I., Mir, R., Almutairi, F. M. & Abu Duhier, F. M. Cytochrome P450: polymorphisms and roles in cancer, diabetes and atherosclerosis. *Asian Pac. J. Cancer Prev.* **19**, 2057–2070 (2018). 10.22034/APJCP.2018.19.8.2057 PMC6171375 30139042

[8] Stading, R., Gastelum, G., Chu, C., Jiang, W. & Moorthy, B. Molecular mechanisms of pulmonary carcinogenesis by polycyclic aromatic hydrocarbons (PAHs): implications for human lung cancer. *Semin Cancer Biol.* **76**, 3–16 (2021). 10.1016/j.semcancer.2021.07.001 PMC8728691 34242741

[9] Moorthy, B., Chu, C. & Carlin, D. J. Polycyclic aromatic hydrocarbons: from metabolism to lung cancer. *Toxicol. Sci.* **145**, 5–15 (2015). 10.1093/toxsci/kfv040 PMC4408964 25911656

[10] Liu, J., Sridhar, J. & Foroozesh, M. Cytochrome P450 family 1 inhibitors and structure-activity relationships. *Molecules* **18**, 14470–14495 (2013). 10.3390/molecules181214470 PMC4216474 24287985

[11] Wesley, S. R., Gallo, M., Apata, T., Van Dis, J. & Hollenbach, S. J. Impact of endocrine-disrupting chemicals, climate, and air pollution on pregnancy outcomes: a scoping review. *Semin Reprod. Med*. **42**, 312–329 (2024). 10.1055/s-0044-1800961 39694048

[12] Saleem, A., Awan, T. & Akhtar, M. F. A comprehensive review on endocrine toxicity of gaseous components and particulate matter in smog. *Front. Endocrinol. (Lausanne)* **15**. 10.3389/fendo.2024.1294205 (2024). PMC10863453 38352708

[13] Wierzba, W., Radowicki, S., Bojar, I. & Pinkas, J. Effects of environmental pollution with aromatic hydrocarbons on endocrine and metabolic functions of the human placenta. *Ann. Agric. Environ. Med.* **25**, 157–161 (2018). 10.26444/aaem/74406 29575858

[14] Talia, C. et al. Disruption of estrogenic and androgenic bioactivities in human fetuses exposed to maternal smoking. *J. Endocr. Soc.* **5**, A496 (2021).

[15] Waring, R. H., Harris, R. M. & Mitchell, S. C. In utero exposure to carcinogens: epigenetics, developmental disruption and consequences in later life. *Maturitas* **86**, 59–63 (2016). 10.1016/j.maturitas.2016.01.008 26921930

[16] Suter, M. et al. Epigenetic modifiers are altered with increased levels of polycyclic aromatic hydrocarbons (PAHs). *Am. J. Obstet. Gynecol.* **228**, S364–S365 (2023).

[17] Suter, M., Banerjee, S., Huang, S., Moorthy, B. & Aagaard, K. M. Placental epigenetic changes associated with polycyclic aromatic hydrocarbon exposures. *Am. J. Obstet. Gynecol.* **226**, S568–S569 (2022).

[18] Mo, J. et al. Benzo[a]pyrene osteotoxicity and the regulatory roles of genetic and epigenetic factors: a review. *Crit. Rev. Environ. Sci. Technol.* **52**, 3244–3282 (2022).

[19] Loftus, C. T., et al. Maternal exposure to urinary polycyclic aromatic hydrocarbons (PAH) in pregnancy and childhood asthma in a pooled multi-cohort study. *Environ. Int*. **170**. 10.1016/j.envint.2022.107494 (2022). PMC9810359 36279735

[20] Suter, M. A. et al. Association between elevated placental polycyclic aromatic hydrocarbons (PAHs) and PAH-DNA adducts from Superfund sites in Harris County, and increased risk of preterm birth (PTB). *Biochem. Biophys. Res. Commun.* **516**, 344–349 (2019). 10.1016/j.bbrc.2019.06.049 PMC6637943 31208719

[21] Tang, D. et al. PAH-DNA adducts in cord blood and fetal and child development in a Chinese cohort. *Environ. Health Perspect.* **114**, 1297–1300 (2006). 10.1289/ehp.8939 PMC1552014 16882543

[22] Perera, F. P. et al. Effects of transplacental exposure to environmental pollutants on birth outcomes in a multiethnic population. *Environ. Health Perspect.* **111**, 201–205 (2003). 10.1289/ehp.5742 PMC1241351 12573906

[23] Doherty, B. T. et al. Chemical exposures assessed via silicone wristbands and endogenous plasma metabolomics during pregnancy. *J. Expo. Sci. Environ. Epidemiol.* **32**, 259–267 (2022). 10.1038/s41370-021-00394-6 PMC8930423 34702988

[24] Maciel-Ruiz, J. A. et al. Geographical approach analysis of the impact of air pollution on newborn intrauterine growth and cord blood DNA damage in Mexico City. *J. Expo. Sci. Environ. Epidemiol.* **34**, 907–916 (2024). 10.1038/s41370-023-00618-x PMC11446826 38086972

[25] Arksey, H. & O’Malley, L. Scoping studies: towards a methodological framework. *Int. J. Soc. Res. Methodol.* **8**, 19–32 (2005).

[26] Montano, L. et al. Polycyclic aromatic hydrocarbons (PAHs) in the environment: occupational exposure, health risks and fertility implications. *Toxics*. **13**. 10.3390/toxics13030151 (2025). PMC11946043 40137477

[27] Feichtinger, W., Papalambrou, K., Poehl, M., Krischker, U. & Neumann, K. Smoking and in vitro fertilization: a meta-analysis. *J. Assist Reprod. Genet* **14**, 596–599 (1997). 10.1023/A:1022584802711 PMC3454733 9447461

[28] Esakky, P. & Moley, K. H. Paternal smoking and germ cell death: a mechanistic link to the effects of cigarette smoke on spermatogenesis and possible long-term sequelae in offspring. *Mol. Cell Endocrinol.* **435**, 85–93 (2016). 10.1016/j.mce.2016.07.015 PMC5014701 27424142

[29] Li, P. et al. Polycyclic aromatic hydrocarbons exposure and early miscarriage in women undergoing in vitro fertilization-embryo transfer. *Hum. Fertil.* **23**, 17–22 (2020). 10.1080/14647273.2018.1479888 29950120

[30] Neal, M. S., Zhu, J. & Foster, W. G. Quantification of benzo[a]pyrene and other PAHs in the serum and follicular fluid of smokers versus non-smokers. *Reprod. Toxicol.* **25**, 100–106 (2008). 10.1016/j.reprotox.2007.10.012 18065195

[31] Younglai, E. V., Holloway, A. C. & Foster, W. G. Environmental and occupational factors affecting fertility and IVF success. *Hum. Reprod. Update* **11**, 43–57 (2005). 10.1093/humupd/dmh055 15601728

[32] Young, A. S. et al. Integrated chemical exposome–metabolome profiling of follicular fluid and associations with fertility outcomes during assisted reproduction. *Environ Int*. **203**. 10.1016/j.envint.2025.109787 (2025). PMC12720341 40946429

[33] Raimondo, S. et al. Comparative analysis of the bioaccumulation of bisphenol A in the blood serum and follicular fluid of women living in two areas with different environmental impacts. *Front Endocrinol. (Lausanne)* **15**. 10.3389/FENDO.2024.1392550 (2024). PMC11495266 39439569

[34] Ferrero, G. et al. Small noncoding RNAs and sperm nuclear basic proteins reflect the environmental impact on germ cells. *Mol Med*. **30**. 10.1186/S10020-023-00776-6 (2024). PMC10799426 38243211

[35] Saad, A. A. et al. Effect of polycyclic aromatic hydrocarbons exposure on sperm DNA in idiopathic male infertility. *J Health Pollut*. **9** 10.5696/2156-9614-9.21.190309 (2019). PMC6421954 30931169

[36] Yang, P. et al. Mediation of association between polycyclic aromatic hydrocarbon exposure and semen quality by spermatogenesis-related microRNAs: a pilot study in an infertility clinic. *J. Hazard Mater*. **384**. 10.1016/j.jhazmat.2019.121431 (2020). 31672436

[37] Capone, S. et al. Innovative approach for human semen quality assessment based on volatilomics. *Toxics* **12** 10.3390/TOXICS12080543 (2024). PMC11360181 39195645

[38] Rossner, P. et al. Oxidative damage to placental DNA, air pollution, genetic polymorphisms and pregnancy outcomes. *Cancer Res*. **70**. 10.1158/1538-7445.AM10-4392 (2010).

[39] Tsinganou, E. & Gebbers, J. O. Reproductive health and air pollution. *Gynakol. Prax.* **33**, 591–596 (2009).

[40] Sram, R. J. Impact of air pollution on reproductive health. *Environ. Health Perspect.* **107**, A542–A543 (1999). 10.1289/ehp.107-1566709 PMC1566709 10627171

[41] Shah, P. S. & Balkhair, T. Knowledge Synth Grp Determinants P. Air pollution and birth outcomes: a systematic review. *Environ. Int.* **37**, 498–516 (2011). 10.1016/j.envint.2010.10.009 21112090

[42] Proietti, E., Röösli, M., Frey, U. & Latzin, P. Air pollution during pregnancy and neonatal outcome: a review. *J. Aerosol Med Pulm. Drug Deliv.* **26**, 9–23 (2013). 10.1089/jamp.2011.0932 22856675

[43] Laaidi, M. et al. Air pollution and pregnancy outcome: a review of the literature. *Environ. Risques Sante* **10**, 287–298 (2011).

[44] Polichetti, G. et al. Effects of ambient air pollution on birth outcomes: an overview. *Crit. Rev. Environ. Sci. Technol.* **43**, 1223–1245 (2013).

[45] Šrám, R. J. et al. Teplice Program - The impact of air pollution on human health. *Environ. Health Perspect.* **104**, 699–714 (1996). 10.1289/ehp.104-1469669 PMC1469669 8879999

[46] Srám, R. J., Binková, B. B., Dejmek, J. & Bobak, M. Ambient air pollution and pregnancy outcomes: a review of the literature. *Environ. Health Perspect.* **113**, 375–382 (2005). 10.1289/ehp.6362 PMC1278474 15811825

[47] Pedersen, M. Is it still important to study if ambient air pollution triggers stillbirth? *Occup. Environ. Med*. **73**, 571–572 (2016). 10.1136/oemed-2016-103625 27221105

[48] Liu, X. X. et al. Polycyclic aromatic hydrocarbons exposure as a potential risk factor for miscarriage among women in the United States: a secondary dataset analysis of NHANES data for the period 2005-2014. *Tob. Induc. Dis*. **23**. 10.18332/tid/205903 (2025). PMC12332853 40785795

[49] Ussher, M. et al. Toxicant and nicotine exposure in pregnant smokers, vapers, and nicotine-replacement users: cross-sectional study. *Nicotine Tob. Res*. 10.1093/ntr/ntaf103 (2025). PMC12453684 40576585

[50] Xie, C. B. et al. Combinations of CYP2A6*4 and glutathione S-transferases gene polymorphisms modify the association between maternal secondhand smoke exposure during pregnancy and small-for-gestational-age. *Nicotine Tob. Res.* **17**, 1421–1427 (2015). 10.1093/ntr/ntv072 25847289

[51] Dai, Y. F., Xu, X. J., Huo, X. & Faas, M. M. Effects of polycyclic aromatic hydrocarbons (PAHs) on pregnancy, placenta, and placental trophoblasts. *Ecotoxicol. Environ. Saf*. **262**. 10.1016/j.ecoenv.2023.115314 (2023). 37536008

[52] Gladen, B. C. et al. Polycyclic aromatic hydrocarbons in placenta. *Hum. Exp. Toxicol.* **19**, 597–603 (2000). 10.1191/096032700671433928 11211237

[53] Gustavsson, P. et al. Risk of preeclampsia and gestational diabetes after occupational exposure to chemicals during pregnancy–A cohort study of births in Sweden 1994–2014. *Environ. Res*. **279**. 10.1016/j.envres.2025.121802 (2025). 40345419

[54] Nazarpour, S. et al. Investigation of the relationship between air pollution and gestational diabetes. *J. Obstet. Gynaecol. (Lahore)* **44**, 2362962 (2024). 10.1080/01443615.2024.2362962 38853776

[55] Malmqvist, E., Jakobsson, K., Tinnerberg, H., Rignell-Hydbom, A. & Rylander, L. Gestational diabetes and preeclampsia in association with air pollution at levels below current air quality guidelines. *Environ. Health Perspect.* **121**, 488–493 (2013). 10.1289/ehp.1205736 PMC3620758 23563048

[56] Pedersen, M. et al. Impact of road traffic pollution on pre-eclampsia and pregnancy-induced hypertensive disorders. *Epidemiology* **28**, 99–106 (2017). 10.1097/EDE.0000000000000555 PMC5380106 27648591

[57] Luo, L. et al. Single and joint associations of exposure to polycyclic aromatic hydrocarbons with blood coagulation function during pregnancy: a cross-sectional study. *Sci. Total Environ.* **885**, 10.1016/j.scitotenv.2023.163949 (2023). 37149174

[58] Smarr, M. M. et al. Persistent organic pollutants and pregnancy complications. *Sci. Total Environ.* **551**, 285–291 (2016). 10.1016/j.scitotenv.2016.02.030 PMC7751424 26878640

[59] Wang, X. et al. Systemic oxidative stress levels during the course of pregnancy: Associations with exposure to air pollutants. *Environ. Pollut*. **357**. 10.1016/j.envpol.2024.124463 (2024). PMC11418402 38942277

[60] Wu, J. et al. Association between local traffic-generated air pollution and preeclampsia and preterm delivery in the South Coast Air Basin of California. *Environ. Health Perspect.* **117**, 1773–1779 (2009). 10.1289/ehp.0800334 PMC2801174 20049131

[61] Zhen, H. et al. Associations of polycyclic aromatic hydrocarbons exposure with perinatal anxiety symptoms. *BMC Public Health* **25**, 1245 (2025). 10.1186/s12889-025-22424-w PMC11967036 40175925

[62] Porpora, M. G. et al. Environmental contaminants exposure and preterm birth: a systematic review. *Toxics*. **7**. 10.3390/toxics7010011 (2019). PMC6468584 30832205

[63] Suter, M., Moorthy, B. & Aagaard, K. Targeted mass spectrometry of the placenta reveals exposure to polycyclic aromatic hydrocarbons (PAHs) is associated with gestational age at birth. *Am. J. Obstet. Gynecol.* **216**, S357 (2017).

[64] Hummel, G. et al. Placental polycyclic aromatic hydrocarbon (PAH) levels are associated with spontaneous preterm birth. *Int. J. Mol. Sci*. **26**. 10.3390/ijms26178179 (2025). PMC12428254 40943106

[65] Agarwal, P., Singh, L., Anand, M. & Taneja, A. Association between placental polycyclic aromatic hydrocarbons (PAHs), oxidative stress, and preterm delivery: a case–control study. *Arch. Environ. Contam Toxicol.* **74**, 218–227 (2018). 10.1007/s00244-017-0455-0 28916946

[66] Choi, H., Rauh, V., Garfinkel, R., Tu, Y. & Perera, F. P. Prenatal exposure to airborne polycyclic aromatic hydrocarbons and risk of intrauterine growth restriction. *Environ. Health Perspect.* **116**, 658–665 (2008). 10.1289/ehp.10958 PMC2367680 18470316

[67] Perera, F. P. et al. Molecular evidence of an interaction between prenatal environmental exposures and birth outcomes in a multiethnic population. *Environ. Health Perspect.* **112**, 626–630 (2004). 10.1289/ehp.6617 PMC1241932 15064172

[68] Padula, A. M. et al. Exposure to airborne polycyclic aromatic hydrocarbons during pregnancy and risk of preterm birth. *Environ. Res.* **135**, 221–226 (2014). 10.1016/j.envres.2014.09.014 PMC4262545 25282280

[69] Liu, X. C., Strodl, E., Huang, L. H., Hu, B. J. & Chen, W. Q. Effect of prenatal exposure to household air pollution from multiple sources on risk of preterm birth. *Atmosphere (Basel)* **13**. 10.3390/atmos13122022 (2022).

[70] Wing, S. E. et al. Preterm birth among infants exposed to in utero ultrafine particles from aircraft emissions. *Environ. Health Perspect*. **128**. 10.1289/EHP5732 (2020). PMC7228090 32238012

[71] Zhao, N. et al. Effects of Benzo[a]pyrene-DNA adducts, dietary vitamins, folate, and carotene intakes on preterm birth: a nested case-control study from the birth cohort in China. *Environ. Health* **21**. 10.1186/s12940-022-00859-7 (2022). PMC9074263 35513839

[72] Barrett, E. S. et al. Prenatal polycyclic aromatic hydrocarbon (PAH) exposure in relation to placental corticotropin releasing hormone (pCRH) in the CANDLE pregnancy cohort. *Front Endocrinol. (Lausanne)* **13**, 1011689 (2022). 10.3389/fendo.2022.1011689 PMC9691680 36440232

[73] Cathey, A. L. et al. Associations between urinary hydroxylated polycyclic aromatic hydrocarbon biomarker concentrations and measures of timing of delivery and infant size at birth. *Environ. Int*. **190**. 10.1016/j.envint.2024.108848 (2024). PMC12912189 38936064

[74] Freije, S. L. et al. Prenatal exposure to polycyclic aromatic hydrocarbons and gestational age at birth. *Environ. Int*. **164**, 107246 (2022). 10.1016/j.envint.2022.107246 PMC9269995 35453081

[75] Huel, G. et al. Aryl hydrocarbon hydroxylase activity in human placenta and threatened preterm delivery. *J. Expo. Anal. Environ. Epidemiol.* **3**, 187–199 (1993). 9857304

[76] Balalian, A. A., McVeigh, K. H., Stingone, J. A. Air pollution, children’s academic achievement and the potential mediating role of preterm birth. *Int. J. Hyg. Environ. Health* **243**. 10.1016/j.ijheh.2022.113991 (2022). PMC9541921 35688002

[77] Ferguson, K. K., O’Neill, M. S. & Meeker, J. D. Environmental contaminant exposures and preterm birth: a comprehensive review. *J. Toxicol. Environ. Health B Crit. Rev.* **16**, 69–113 (2013). 10.1080/10937404.2013.775048 PMC3889157 23682677

[78] Bosetti, C. et al. Ambient particulate matter and preterm birth or birth weight: a review of the literature. *Arch. Toxicol.* **84**, 447–460 (2010). 10.1007/s00204-010-0514-z 20140425

[79] Niwa, M., Suzuki, Y., Yoshinaga, J., Watanabe, C. & Mizumoto, Y. Prenatal exposure to polycyclic aromatic hydrocarbons and birth outcomes. *Polycycl. Aroma Compd.* **31**, 16–27 (2011).

[80] Al-Saleh, I. et al. Polycyclic aromatic hydrocarbons (PAHs) as determinants of various anthropometric measures of birth outcome. *Sci. Total Environ.* **444**, 565–578 (2013). 10.1016/j.scitotenv.2012.12.021 23314068

[81] Langlois, P. H. et al. Maternal occupational exposure to polycyclic aromatic hydrocarbons and risk of neural tube defect-affected pregnancies. *Birth Defects Res. A Clin. Mol. Teratol.* **94**, 693–700 (2012). 10.1002/bdra.23045 PMC5048886 22807044

[82] Ren, A. et al. Association of selected persistent organic pollutants in the placenta with the risk of neural tube defects. *Proc. Natl. Acad. Sci. USA* **108**, 12770–12775 (2011). 10.1073/pnas.1105209108 PMC3150927 21768370

[83] Wang, B. et al. Levels of polycyclic aromatic hydrocarbons in maternal serum and risk of neural tube defects in offspring. *Environ. Sci. Technol.* **49**, 588–596 (2015). 10.1021/es503990v PMC4291766 25488567

[84] Yi, D. et al. Levels of PAH-DNA adducts in cord blood and cord tissue and the risk of fetal neural tube defects in a Chinese population. *Neurotoxicology* **46**, 73–78 (2015). 10.1016/j.neuro.2014.12.003 PMC4339482 25522656

[85] Langlois, P. H. et al. Maternal occupational exposure to polycyclic aromatic hydrocarbons and risk of oral cleft-affected pregnancies. *Cleft Palate Craniofacial J.* **50**, 337–346 (2013). 10.1597/12-104 PMC4096036 23136939

[86] Ravi, M. B. et al. Influence of domestic exposure to polycyclic aromatic hydrocarbons on women’s periconceptional stage and associated risk of oral cleft in offspring. *J. Pharm. Bioallied Sci.* **14**, 589–594 (2022). 10.4103/jpbs.jpbs_659_21 PMC9469367 36110813

[87] Pi, X. et al. Periconceptional polycyclic aromatic hydrocarbon levels in maternal hair and fetal risk for congenital heart defects. *Ecotoxicol. Environ. Saf*. **286**. 10.1016/j.ecoenv.2024.117251 (2024). 39490106

[88] Moorthy, B., Swanson, L., Hoffman, K., Lingappan, K., Jiang, W. TP072 BRONCHOPULMONARY DYSPLASIA / thematic poster session prenatal exposure to polycyclic aromatic hydrocarbons (PAHs) augments neonatal oxygen-mediated lung injury and alters the gut microbiome in mice: mechanistic role of cytochrome P450 (CYP)1A1, 1A2, and 1B1. Accessed October 24, 2025. https://www.atsjournals.org (2025).

[89] Sanguos, C. L. et al. Non-invasive biomonitoring of infant exposure to environmental organic pollutants in north-western Spain based on hair analysis. Identification of potential sources. *Environ. Pollut.* **339**, 122705 (2023). 10.1016/j.envpol.2023.122705 37827353

[90] Sarumi, O. A., Bamgbose, O., Adebowale, A. & James, A. O. Levels and carcinogenic risk assessment of polycyclic aromatic hydrocarbons in the breast milk of selected lactating mothers in Abeokuta, Nigeria. *J. Environ. Sci. Health A Tox Hazard Subst. Environ. Eng.* **60**, 55–65 (2025). 10.1080/10934529.2025.2494456 40267019

[91] Acharya, N. et al. Polycyclic aromatic hydrocarbons in breast milk of obese vs normal women: infant exposure and risk assessment. *Sci. Total Environ.* **668**, 658–667 (2019). 10.1016/j.scitotenv.2019.02.381 30856574

[92] Li, J. et al. Association of prenatal polycyclic aromatic hydrocarbons exposure, DNA hydroxymethylation, and neurodevelopment at 0 and 2 years of age. *Toxics*. **13**. 10.3390/TOXICS13090726 (2025). PMC12473805 41012347

[93] Puvvula, J. et al. Gestational exposure to environmental chemicals and epigenetic alterations in the placenta and cord blood mononuclear cells. *Epigenet. Commun*. **4**. 10.1186/S43682-024-00027-7 (2024). PMC11217138 38962689

[94] Yang, J., Lu, Z., Liu, Z., Wang, L. & Qiang, M. Methylation of imprinted genes in sperm DNA correlated to urinary polycyclic aromatic hydrocarbons (PAHs) exposure levels in reproductive-aged men and the birth outcomes of the offspring. *Front Genet*. **11**. 10.3389/FGENE.2020.611276 (2021) PMC7834272 33505432

[95] Yan Z. et al. Prenatal polycyclic aromatic hydrocarbon, adiposity, peroxisome proliferator-activated receptor (PPAR) γ methylation in offspring, grand-offspring mice. *PLoS One* **9**, 10.1371/JOURNAL.PONE.0110706 (2014). PMC4210202 25347678

[96] Yang, L. et al. The association between prenatal exposure to polycyclic aromatic hydrocarbons and birth weight: a meta-analysis. Published online. 10.1371/journal.pone.0236708 (2020). PMC7425945 32790684

[97] Moghaddamhosseini, V. et al. The association between exposure to polycyclic aromatic hydrocarbons and birth outcomes: a systematic review and meta-analysis of observational studies. *Sci. Total Environ*. **905**. 10.1016/j.scitotenv.2023.166922 (2023). 37699478

[98] Polańska, K., Hanke, W., Sobala, W., Brzeźnicki, S. & Ligocka, D. Exposure to polycyclic aromatic hydrocarbons and newborn biometric indicators. *Int. J. Occup. Med Environ. Health* **23**, 339–346 (2010). 10.2478/v10001-010-0028-1 21306979

[99] Agarwal, P. et al. Placental levels of polycyclic aromatic hydrocarbons (PAHs) and their association with birth weight of infants. *Drug Chem. Toxicol.* **45**, 868–877 (2022). 10.1080/01480545.2020.1783285 32597233

[100] Hu, P. A. et al. Placental weight mediates association between prenatal exposure to cooking oil fumes and preterm birth. *J. Maternal Fetal Neonatal Med.* **35**, 7248–7258 (2022). 10.1080/14767058.2021.1946783 34219591

[101] Kumar, S. N. et al. Predicting risk of low birth weight offspring from maternal features and blood polycyclic aromatic hydrocarbon concentration. *Reprod. Toxicol.* **94**, 92–100 (2020). 10.1016/j.reprotox.2020.03.009 32283251

[102] Snijder, C. A. et al. Occupational exposure to chemicals and fetal growth: the Generation R Study. *Hum. Reprod.* **27**, 910–920 (2012). 10.1093/humrep/der437 PMC3279127 22215632

[103] Sram, R. J. et al. Association of DNA adducts and genotypes with birth weight. *Mutat. Res Genet Toxicol. Environ. Mutagen* **608**, 121–128 (2006). 10.1016/j.mrgentox.2006.04.022 16837240

[104] Dejmek, J. et al. Exposure to carcinogenic PAHs in utero and fetal growth. *Epidemiology* **10**, S126–S126 (1999).

[105] Sram, R. J. et al. Prenatal exposure to polycyclic aromatic hydrocarbons and growth parameters. *J. Appl. Biomed.* **22**, 12–22 (2024). 10.32725/jab.2024.004 38505966

[106] Cao, C. et al. Prenatal exposure to polycyclic aromatic hydrocarbons could increase the risk of low birth weight by affecting the DNA methylation states in a Chinese cohort. *Reprod. Biol.* **21**, 100574 (2021). 10.1016/j.repbio.2021.100574 34794034

[107] Choi, H. Prenatal exposure to PAHs and fetal growth restriction in a minority cohort. *Epidemiology* **18**, S100–S100 (2007).

[108] Jiang, L. et al. Association between fetal exposure to polycyclic aromatic hydrocarbons and low birth weight: a case-control study in Shenzhen, China. *Environ. Sci. Pollut. Res. Int.* **29**, 88779–88787 (2022). 10.1007/s11356-022-21965-6 35840835

[109] Li, X. et al. Maternal urinary levels of PAH metabolites, umbilical cord blood telomere length and anthropometric indices in newborns. *Ecotoxicol. Environ. Saf.* **291**, 117767 (2025). 10.1016/j.ecoenv.2025.117767 39874713

[110] Pedersen, M. et al. Bulky DNA adducts in cord blood, maternal fruit-and-vegetable consumption, and birth weight in a European mother-child study (NewGeneris). *Environ. Health Perspect.* **121**, 1200–1206 (2013). 10.1289/ehp.1206333 PMC3801201 23906905

[111] Perera, F. P. et al. Relationship between polycyclic aromatic hydrocarbon-DNA adducts, environmental tobacco smoke, and child development in the World Trade Center cohort. *Environ. Health Perspect.* **115**, 1497–1502 (2007). 10.1289/ehp.10144 PMC2022637 17938742

[112] Álvarez-Silvares, E. et al. Determination of organic pollutants in meconium and its relationship with fetal growth. Case control study in Northwestern Spain. *J. Perinat. Med*. **49**, 884–896 (2021). 10.1515/jpm-2020-0324 33856139

[113] Keerthy, D. et al. The effect of in utero polycyclic aromatic hydrocarbon exposure on the neonatal meconium microbiome. *J. Investig. Med.* **70**, 1069–1070 (2022). 10.1016/j.envres.2024.120053 PMC11816390 39341532

[114] Keerthy, D. et al. An evaluation of in utero polycyclic aromatic hydrocarbon exposure on the neonatal meconium microbiome. *Environ. Res.* **263**, 120053 (2024). 10.1016/j.envres.2024.120053 PMC11816390 39341532

[115] Duarte-Salles, T., Mendez, M. A., Meltzer, H. M., Alexander, J. & Haugen, M. Dietary benzo(a)pyrene intake during pregnancy and birth weight: associations modified by vitamin C intakes in the Norwegian Mother and Child Cohort Study (MoBa). *Environ. Int*. **60**, 217–223 (2013). 10.1016/j.envint.2013.08.016 24071023

[116] Duarte-Salles, T. et al. Dietary benzo(a)pyrene and fetal growth: effect modification by vitamin C intake and glutathione S-transferase P1 polymorphism. *Environ. Int.* **45**, 1–8 (2012). 10.1016/j.envint.2012.04.002 PMC3855239 22565211

[117] Lamichhane, D. K. et al. Impact of prenatal exposure to polycyclic aromatic hydrocarbons from maternal diet on birth outcomes: a birth cohort study in Korea. *Public Health Nutr.* **19**, 2562–2571 (2016). 10.1017/S1368980016000550 PMC10271019 27056033

[118] Sewor, C., Eliason, S., Jaakkola, J. J. K., Amegah, A. K. Dietary polycyclic aromatic hydrocarbon (PAH) consumption and risk of adverse birth outcomes: a systematic review and meta-analysis. *Environ. Health Perspect*. **132**. 10.1289/EHP12922 (2024). PMC10798428 38241191

[119] Ardiyani, V., Kelly, F., Phillips, D., Wooster, M., Grosvernor, M. Reflection of prenatal exposure to wildfire smoke on reduced birth weight in 2015–2019. *7th Environmental Technology and Management Conference, ETMC 2023.* Vol. 485. 10.1051/e3sconf/202448507010 (2024).

[120] Arinola, G. O., Dutta, A., Oluwole, O., Olopade, C. O. Household air pollution, levels of micronutrients and heavy metals in cord and maternal blood, and pregnancy outcomes. *Int. J. Environ. Res. Public Health*. **15**. 10.3390/ijerph15122891 (2018). PMC6313792 30562990

[121] Barn, P. et al. Coal smoke, gestational cadmium exposure, and fetal growth. *Environ Res*. **179**. 10.1016/j.envres.2019.108830 (2019). 31678728

[122] Gong, X., Lin, Y. & Zhan, F. B. Industrial air pollution and low birth weight: a case-control study in Texas, USA. *Environ. Sci. Pollut. Res.* **25**, 30375–30389 (2018). 10.1007/s11356-018-2941-y 30159842

[123] Gong, X., Lin, Y., Bell, M. L. & Zhan, F. B. Associations between maternal residential proximity to air emissions from industrial facilities and low birth weight in Texas, USA. *Environ. Int.* **120**, 181–198 (2018). 10.1016/j.envint.2018.07.045 30096612

[124] Veber, T., Dahal, U., Lang, K., Orru, K. & Orru, H. Industrial air pollution leads to adverse birth outcomes: a systematized review of different exposure metrics and health effects in newborns. *Public Health Rev*. **43**. 10.3389/phrs.2022.1604775 (2022). PMC9400407 36035982

[125] Van Buren, K. W. et al. Maternal occupational exposure to selected organic and chlorinated solvents and delivery of small-for-gestational age or preterm infants. *Am. J. Ind. Med*. **66**, 842–853 (2023). 10.1002/ajim.23516 PMC10527887 37463847

[126] Birks, L. et al. Occupational exposure to endocrine-disrupting chemicals and birth weight and length of gestation: a European meta-analysis. *Environ. Health Perspect.* **124**, 1785–1793 (2016). 10.1289/EHP208 PMC5089886 27152464

[127] Glinianaia, S. V., Rankin, J., Bell, R., Pless-Mulloli, T. & Howel, D. Particulate air pollution and fetal health a systematic review of the epidemiologic evidence. *Epidemiology* **15**, 36–45 (2004). 10.1097/01.ede.0000101023.41844.ac 14712145

[128] Ritz, B. & Wilhelm, M. Ambient air pollution and adverse birth outcomes: methodologic issues in an emerging field. *Basic Clin. Pharm. Toxicol.* **102**, 182–190 (2008). 10.1111/j.1742-7843.2007.00161.x PMC3656653 18226073

[129] Lu, C. et al. Effect of outdoor air pollution and indoor environmental factors on small for gestational age. *Build Environ*. **206**. 10.1016/j.buildenv.2021.108399 (2021).

[130] Ritz, B. et al. Prenatal air pollution exposure and ultrasound measures of fetal growth in Los Angeles, California. *Environ. Res*. **130**, 7–13 (2014). 10.1016/j.envres.2014.01.006 PMC4016959 24517884

[131] Syama, K. P., Blais, E., Kumarathasan, P. Maternal mechanisms in air pollution exposure-related adverse pregnancy outcomes: a systematic review. *Sci. Total Environ.* **970**. 10.1016/j.scitotenv.2025.178999 (2025). 40043646

[132] Ballester, F. et al. Air pollution exposure during pregnancy and reduced birth size: a prospective birth cohort study in Valencia, Spain. *Environ. Health*. **9**. 10.1186/1476-069X-9-6 (2010). PMC2845572 20113501

[133] Choi, H., Wang, L., Lin, X., Spengler, J. D. & Perera, F. P. Fetal window of vulnerability to airborne polycyclic aromatic hydrocarbons on proportional intrauterine growth restriction. *PLoS One* **7**, e35464 (2012). 10.1371/journal.pone.0035464 PMC3335852 22545107

[134] Jedrychowski, W. et al. Gender differences in fetal growth of newborns exposed prenatally to airborne fine particulate matter. *Environ. Res.* **109**, 447–456 (2009). 10.1016/j.envres.2009.01.009 PMC3786262 19261271

[135] Ghosh, R., Rankin, J., Pless-Mulloli, T. & Glinianaia, S. Does the effect of air pollution on pregnancy outcomes differ by gender? A systematic review. *Environ. Res*. **105**, 400–408 (2007). 10.1016/j.envres.2007.03.009 17493608

[136] Suzuki, Y. et al. Prenatal exposure to phthalate esters and PAHs and birth outcomes. *Environ. Int.* **36**, 699–704 (2010). 10.1016/j.envint.2010.05.003 20605637

[137] Maypole-Keenan, C. M., Symanski, E., Stock, T. H. & Waller, D. K. Association between ambient levels of polycyclic aromatic hydrocarbons and small for gestational age hispanic infants born along the United States-Mexico border. *J. Immigr. Minor Health* **18**, 143–149 (2016). 10.1007/s10903-014-9994-x PMC11522966 24585213

[138] Sampaio, G. R. et al. Polycyclic aromatic hydrocarbons in foods: biological effects, legislation, occurrence, analytical methods, and strategies to reduce their formation. *Int. J. Mol. Sci*. **22**. 10.3390/IJMS22116010 (2021). PMC8199595 34199457

[139] Choi, H. & Perera, F. P. Sources of greater fetal vulnerability to airborne polycyclic aromatic hydrocarbons among African Americans. *J. Epidemiol. Community Health (1978)* **66**, 121–126 (2012). 10.1136/jech.2009.099051 PMC3201730 20805195

[140] Lupo, P. J. et al. Maternal occupational exposure to polycyclic aromatic hydrocarbons: Effects on gastroschisis among offspring in the National Birth Defects Prevention Study. *Environ. Health Perspect.* **120**, 910–915 (2012). 10.1289/ehp.1104305 PMC3385431 22330681

[141] Santiago-Colón, A. et al. Association between maternal occupational exposure to polycyclic aromatic hydrocarbons and rare birth defects of the face and central nervous system. *Birth Defects Res.* **112**, 404–417 (2020). 10.1002/bdr2.1643 PMC8641638 31944002

[142] O’Brien, J. L. et al. Maternal occupational exposure to polycyclic aromatic hydrocarbons and craniosynostosis among offspring in the National Birth Defects Prevention Study. *Birth Defects Res. A Clin. Mol. Teratol.* **106**, 55–60 (2016). 10.1002/bdra.23389 PMC4668225 26033890

[143] Van Den Hazel, P. et al. Today’s epidemics in children: possible relations to environmental pollution and suggested preventive measures. *Acta Paediatr. Int. J. Paediatr.* **95**, 18–25 (2006). 10.1080/08035320600885846 17000565

[144] Burton, A. Coal fire emissions curb children’s growth. *Environ. Health Perspect.* **119**, A246–A247 (2011). 10.1289/ehp.119-a246a PMC3114835 21628123

[145] Burton, A. Children’s health: methylation links prenatal PAH exposure to asthma. *Environ. Health Perspect.* **117**, A195 (2009). 10.1289/ehp.117-a195 PMC2685862 19478982

[146] Cordier, S. et al. Parental exposure to polycyclic aromatic hydrocarbons and the risk of childhood brain tumors: the SEARCH International Childhood Brain Tumor Study. *Am. J. Epidemiol.* **159**, 1109–1116 (2004). 10.1093/aje/kwh154 15191928

[147] Ghosh, R. et al. Air pollutants, genes and early childhood acute bronchitis. *Mutat. Res.* **749**, 80–86 (2013). 10.1016/j.mrfmmm.2013.04.001 23648357

[148] Hertz-Picciotto, I. et al. Early childhood lower respiratory illness and air pollution. *Environ. Health Perspect.* **115**, 1510–1518 (2007). 10.1289/ehp.9617 PMC2022654 17938744

[149] Tang, D. L. et al. Effects of prenatal exposure to coal-burning pollutants on children’s development in China. *Environ. Health Perspect.* **116**, 674–679 (2008). 10.1289/ehp.10471 PMC2367664 18470301

[150] Wallace, E. R. et al. Prenatal exposure to polycyclic aromatic hydrocarbons is not associated with behavior problems in preschool and early school-aged children: a prospective multi-cohort study. *Environ Res*. **216**. 10.1016/j.envres.2022.114759 (2023). PMC9817935 36370819

[151] Gheissari, R. et al. Health outcomes in children associated with prenatal and early-life exposures to air pollution: a narrative review. *Toxics* **10**, 458 (2022). 10.3390/toxics10080458 PMC9415268 36006137

[152] Jerzynska, J. et al. Prenatal and postnatal exposure to polycyclic aromatic hydrocarbons and allergy symptoms in city children. *Allergol. Immunopathol. (Madr.* **45**, 18–24 (2017). 10.1016/j.aller.2016.07.006 27789067

[153] Jedrychowski, W. A. et al. Separate and joint effects of tranplacental and postnatal inhalatory exposure to polycyclic aromatic hydrocarbons: prospective birth cohort study on wheezing events. *Pediatr. Pulmonol.* **49**, 162–172 (2014). 10.1002/ppul.22923 PMC4021044 24155203

[154] Jedrychowski, W. et al. Prenatal ambient air exposure to polycyclic aromatic hydrocarbons and the occurrence of respiratory symptoms over the first year of life. *Eur. J. Epidemiol.* **20**, 775–782 (2005). 10.1007/s10654-005-1048-1 16170661

[155] Jedrychowski, W. A. et al. Effect of prenatal exposure to fine particulate matter on ventilatory lung function of preschool children of non-smoking mothers. *Paediatr. Perinat. Epidemiol.* **24**, 492–501 (2010). 10.1111/j.1365-3016.2010.01136.x PMC3761386 20670230

[156] Miller, R. L. et al. Polycyclic aromatic hydrocarbons, environmental tobacco smoke, and respiratory symptoms in an inner-city birth cohort. *Chest* **126**, 1071–1078 (2004). 10.1378/chest.126.4.1071 PMC2223076 15486366

[157] Jedrychowski, W. A. et al. Long term effects of prenatal and postnatal airborne PAH exposures on ventilatory lung function of non-asthmatic preadolescent children. Prospective birth cohort study in Krakow. *Sci. Total Environ.* **502**, 502–509 (2015). 10.1016/j.scitotenv.2014.09.051 PMC4254060 25300014

[158] Zhen, H. et al. Association of polycyclic aromatic hydrocarbons exposure with child neurodevelopment and adult emotional disorders: A meta-analysis study. *Ecotoxicol. Environ. Saf.* **255**, 114770 (2023). 10.1016/j.ecoenv.2023.114770 36931089

[159] Jedrychowski, W. A. et al. Prenatal exposure to polycyclic aromatic hydrocarbons and cognitive dysfunction in children. *Environ. Sci. Pollut. Res. Int.* **22**, 3631–3639 (2015). 10.1007/s11356-014-3627-8 PMC4334078 25253062

[160] Perera, F., Weiland, K., Neidell, M. & Wang, S. Prenatal exposure to airborne polycyclic aromatic hydrocarbons and IQ: estimated benefit of pollution reduction. *J. Public Health Policy* **35**, 327–336 (2014). 10.1057/jphp.2014.14 PMC4231817 24804951

[161] Perera, F. P. et al. Prenatal airborne polycyclic aromatic hydrocarbon exposure and child IQ at age 5 years. *Pediatrics* **124**, e195–e202 (2009). 10.1542/peds.2008-3506 PMC2864932 19620194

[162] Li, Z. et al. Polycyclic aromatic hydrocarbons exposure in early pregnancy on child neurodevelopment. *Environ. Pollut.* **366**, 125527 (2025). 10.1016/j.envpol.2024.125527 39675657

[163] Perera, F. P. et al. Combined effects of prenatal exposure to polycyclic aromatic hydrocarbons and material hardship on child ADHD behavior problems. *Environ. Res*. **160**, 506–513 (2018). 10.1016/j.envres.2017.09.002 PMC5724364 28987706

[164] Chiu, Y. H. M. et al. Prenatal particulate air pollution and neurodevelopment in urban children: examining sensitive windows and sex-specific associations. *Environ. Int*. **87**, 56 (2015). 10.1016/j.envint.2015.11.010 PMC4691396 26641520

[165] Lertxundi, A. et al. Prenatal exposure to PM2.5 and NO2 and sex-dependent infant cognitive and motor development. *Environ. Res.* **174**, 114–121 (2019). 10.1016/j.envres.2019.04.001 31055169

[166] Jedrychowski, W. et al. Effect of prenatal PAH exposure on birth outcomes and neurocognitive development in a cohort of newborns in Poland. Study design and preliminary ambient data. *Int. J. Occup. Med Environ. Health* **16**, 21–29 (2003). 12705714

[167] Peterson, B. S. et al. Effects of prenatal exposure to air pollutants (polycyclic aromatic hydrocarbons) on the development of brain white matter, cognition, and behavior in later childhood. *JAMA Psychiatry* **72**, 531–540 (2015). 10.1001/jamapsychiatry.2015.57 PMC4456286 25807066

[168] Ramphal, B., Herbstman, J. & Margolis, A. Contributions of prenatal exposure to air pollution and childhood housing insecurity to adolescent hippocampal volumes and depression. *Neuropsychopharmacology* **47**, 213 (2022).

[169] Holme, J. A., Myhre, O., & Øvrevik, J. Adverse neurodevelopment in children associated with prenatal exposure to fine particulate matter (PM2.5) – Possible roles of polycyclic aromatic hydrocarbons (PAHs) and mechanisms involved. *Reproduct. Toxicol*. **130**. 10.1016/j.reprotox.2024.108718 (2024). 39276806

[170] Rahman, S. M. et al. Polycyclic aromatic hydrocarbon (PAH) exposure during pregnancy and child anthropometry from birth to 10 years of age: sex-specific evidence from a cohort study in rural Bangladesh. *Environ Res*. **227**. 10.1016/j.envres.2023.115787 (2023). 36997043

[171] Moore, S. et al. The association between ADHD and environmental chemicals—a scoping review. *Int. J. Environ. Res. Public Health* **19**. 10.3390/ijerph19052849 (2022). PMC8910189 35270544

[172] Perera, F. P. et al. Early-life exposure to polycyclic aromatic hydrocarbons and ADHD behavior problems. *PLoS One* **9**, e111670 (2014). 10.1371/journal.pone.0111670 PMC4221082 25372862

[173] Webb, E. et al. Neurodevelopmental and neurological effects of chemicals associated with unconventional oil and natural gas operations and their potential effects on infants and children. *Rev. Environ. Health* **33**, 3–29 (2018). 10.1515/reveh-2017-0008 29068792

[174] Becerra, T. A., Wilhelm, M., Olsen, J., Cockburn, M. & Ritz, B. Ambient air pollution and autism in Los Angeles County, California. *Environ. Health Perspect.* **121**, 380–386 (2013). 10.1289/ehp.1205827 PMC3621187 23249813

[175] Feng, Y. et al. Dyslexia is associated with urinary polycyclic aromatic hydrocarbon metabolite concentrations of children from China: data from the READ program. *Environm. Pollut.* **346**. 10.1016/j.envpol.2024.123538 (2024). 38341065

[176] Nilsen, F. M., Ruiz, J. D. C. & Tulve, N. S. A meta-analysis of stressors from the total environment associated with children’s general cognitive ability. *Int. J. Environ. Res. Public Health* **17**. 10.3390/ijerph17155451 (2020). PMC7432904 32751096

[177] Lovasi, G. S. et al. Neighborhood social context and individual polycyclic aromatic hydrocarbon exposures associated with child cognitive test scores. *J. Child Fam. Stud.* **23**, 785–799 (2014). 10.1007/s10826-013-9731-4 PMC4075963 24994947

[178] Lin, D. et al. Influence of postnatal polycyclic aromatic hydrocarbon exposure on the neurodevelopment of toddlers at the age of 12 months. *Neurotoxicology* **82**, 45–49 (2021). 10.1016/j.neuro.2020.10.013 33137374

[179] Rauh, V. A. et al. Developmental effects of exposure to environmental tobacco smoke and material hardship among inner-city children. *Neurotoxicol. Teratol.* **26**, 373–385 (2004). 10.1016/j.ntt.2004.01.002 PMC3376003 15113599

[180] Khalil, W. J., Akeblersane, M., Khan, A. S., Moin, A. S. M. & Butler, A. E. Environmental pollution and the risk of developing metabolic disorders: obesity and diabetes. *Int. J. Mol. Sci*. **24**. 10.3390/ijms24108870 (2023). PMC10219141 37240215

[181] Svensson, K. et al. Prenatal exposures to mixtures of endocrine disrupting chemicals and children’s weight trajectory up to age 5.5 in the SELMA study. *Sci. Rep.* **11**, 11036 (2021). 10.1038/s41598-021-89846-5 PMC8155069 34040006

[182] Simkova, S., Veleminsky, M. & Sram, R. J. The impact of air pollution to obesity. *Neuro Endocrinol. Lett.* **41**, 146–153 (2020). 33201649

[183] Starling, A. P. et al. Prenatal exposure to traffic and ambient air pollution and infant weight and adiposity: The Healthy Start study. *Environ Res*. **182**. 10.1016/j.envres.2020.109130 (2020). PMC7394733 32069764

[184] Starling, A. P. et al. Ambient air pollution during pregnancy and DNA methylation in umbilical cord blood, with potential mediation of associations with infant adiposity: the Healthy Start study br. *Environ Res*. **214**. 10.1016/j.envres.2022.113881 (2022). PMC10402394 35835166

[185] Lee, K. J. & Choi, K. Non-carcinogenic health outcomes associated with polycyclic aromatic hydrocarbons (PAHs) exposure in humans: an umbrella review. *Expo. Health* **15**, 95–111 (2023).

[186] Kim, J. S. et al. Longitudinal associations of in utero and early life near-roadway air pollution with trajectories of childhood body mass index. *Environm. Health* **17**. 10.1186/s12940-018-0409-7 (2018). PMC6137930 30213262

[187] Jung, K. H. et al. Polycyclic aromatic hydrocarbon exposure, obesity and childhood asthma in an urban cohort. *Environ. Res.* **128**, 35–41 (2014). 10.1016/j.envres.2013.12.002 PMC3912566 24407477

[188] Chen, X. D. et al. Prenatal exposure to polycyclic aromatic hydrocarbons and blood pressure in the early life of children. *Ecotoxicol. Environ Saf*. **291**. 10.1016/j.ecoenv.2025.117830 (2025). 39933235

[189] Kehm, R. D. et al. Associations of prenatal exposure to polycyclic aromatic hydrocarbons with pubertal timing and body composition in adolescent girls: Implications for breast cancer risk. *Environ. Res.* **196**, 110369 (2021). 10.1016/j.envres.2020.110369 PMC8552520 33131678

[190] Humphreys, J. & Valdés Hernández, M. D. C Impact of polycyclic aromatic hydrocarbon exposure on cognitive function and neurodegeneration in humans: a systematic review and meta-analysis. *Front Neurol.* **13**, 1052333 (2023). 10.3389/fneur.2022.1052333 PMC9871581 36703634

