# Supplementary material for: Polycyclic aromatic hydrocarbons and perinatal outcomes: a scoping review
Source: Pediatr Res. 2026 Jul 3;100(4):1576–87. doi: 10.1038/s41390-026-05264-1 (PMC13759308; doi:10.1038/s41390-026-05264-1)
Supplement: Supplementary file 1 [file 41390_2026_5264_MOESM1_ESM.pdf]

Appendix 1 : Search strategy for PAH and perinatal health (September 16, 2025)

PubMed Search Strategy (conducted [insert date]) (1495 after humans, last 20 yrs and infant)

Database: PubMed (National Library of Medicine)
Date of search: 9/16/25
Search string:

("Polycyclic Aromatic Hydrocarbons"[MeSH] OR "polycyclic aromatic hydrocarbon*"[tiab] OR "polynuclear aromatic hydrocarbon*"[tiab] OR PAH[tiab] OR PAHs[tiab] OR "benzo[a]pyrene"[tiab] OR naphthalene[tiab] OR anthracene[tiab] OR phenanthrene[tiab] OR pyrene[tiab] OR "1-hydroxypyrene"[tiab] OR "1-OHP"[tiab])

AND

("Pregnancy Outcome"[MeSH] OR "Pregnancy Complications"[MeSH] OR perinatal[tiab] OR "perinatal outcome*"[tiab] OR "pregnancy outcome*"[tiab] OR birth[tiab] OR "preterm birth"[tiab] OR prematur*[tiab] OR "low birth weight"[tiab] OR LBW[tiab] OR "small for gestational age"[tiab] OR SGA[tiab] OR "intrauterine growth restriction"[tiab] OR IUGR[tiab] OR stillbirth[tiab] OR miscarriage[tiab] OR "congenital anomaly"[tiab])

Embase (Ovid) example translation (syntax differs, uses /exp for MeSH/Emtree and :ti,ab for fields):

'exp polycyclic aromatic hydrocarbon'/exp OR 'polycyclic aromatic hydrocarbon*':ti,ab OR 'polynuclear aromatic hydrocarbon*':ti,ab OR PAH:ti,ab OR PAHs:ti,ab OR 'benzo[a]pyrene':ti,ab OR naphthalene:ti,ab OR anthracene:ti,ab OR phenanthrene:ti,ab OR pyrene:ti,ab OR '1-hydroxypyrene':ti,ab OR '1-OHP':ti,ab

AND

'exp pregnancy outcome'/exp OR 'exp pregnancy complication'/exp OR perinatal:ti,ab OR 'perinatal outcome*':ti,ab OR 'pregnancy outcome*':ti,ab OR birth:ti,ab OR 'preterm birth':ti,ab OR prematur*:ti,ab OR 'low birth weight':ti,ab OR LBW:ti,ab OR 'small for gestational age':ti,ab OR SGA:ti,ab OR 'intrauterine growth restriction':ti,ab OR IUGR:ti,ab OR stillbirth:ti,ab OR miscarriage:ti,ab OR 'congenital anomaly':ti,ab

Web of Science (Core Collection) example translation (use TS= for topic searches, which covers title, abstract, and keywords):

TS=("polycyclic aromatic hydrocarbon*" OR "polynuclear aromatic hydrocarbon*" OR PAH OR PAHs OR "benzo[a]pyrene" OR naphthalene OR anthracene OR phenanthrene OR pyrene OR "1-hydroxypyrene" OR "1-OHP")

AND

TS=("perinatal" OR "perinatal outcome*" OR "pregnancy outcome*" OR birth OR "preterm birth" OR prematur* OR "low birth weight" OR LBW OR "small for gestational age" OR SGA OR "intrauterine growth restriction" OR IUGR OR stillbirth OR miscarriage OR "congenital anomaly")

High precision Embase code (625 articles)

('polycyclic aromatic hydrocarbon'/exp OR 'benzo[a]pyrene':ti,ab OR '1-hydroxypyrene':ti,ab OR 'PAH exposure':ti,ab OR 'urinary PAH':ti,ab)

AND

('pregnancy outcome'/exp OR 'pregnancy complication'/exp OR 'preterm birth':ti,ab OR 'low birth weight':ti,ab OR 'small for gestational age':ti,ab OR stillbirth:ti,ab OR miscarriage:ti,ab OR 'congenital anomaly':ti,ab)

Pubmed high precision strategy

("Polycyclic Aromatic Hydrocarbons"[MeSH] OR "benzo[a]pyrene"[tiab] OR "1-hydroxypyrene"[tiab] OR "PAH exposure"[tiab] OR "urinary PAH"[tiab])

AND

("Pregnancy Outcome"[MeSH] OR "preterm birth"[tiab] OR "low birth weight"[tiab] OR "small for gestational age"[tiab] OR stillbirth[tiab] OR miscarriage[tiab] OR "congenital anomaly"[tiab])

Web of science high precision (348)

TS=("polycyclic aromatic hydrocarbon*" OR "benzo[a]pyrene" OR "1-hydroxypyrene" OR "PAH exposure" OR "urinary PAH")

AND

TS=("pregnancy outcome*" OR "preterm birth" OR "low birth weight" OR "small for gestational age" OR stillbirth OR miscarriage OR "congenital anomaly")

Total – 2464 articles

after deduplication - 2339
